# Human amniotic membrane conditioned medium inhibits proliferation and modulates related microRNAs expression in hepatocarcinoma cells

**DOI:** 10.1038/s41598-019-50648-5

**Published:** 2019-10-02

**Authors:** Rodrigo Riedel, Antonio Pérez-Pérez, Antonio Carmona-Fernández, Mariana Jaime, Roberto Casale, José Luis Dueñas, Pilar Guadix, Víctor Sánchez-Margalet, Cecilia L. Varone, Julieta L. Maymó

**Affiliations:** 1 Universidad de Buenos Aires. CONICET. Instituto de Química Biológica de la Facultad de Ciencias Exactas y Naturales (IQUIBICEN). Ciudad Universitaria Pabellón 2, 4° piso, (1428), Buenos Aires, Argentina; 2 Universidad de Buenos Aires. Facultad de Ciencias Exactas y Naturales. Departamento de Química Biológica. Ciudad Universitaria Pabellón 2, 4° piso, (1428), Buenos Aires, Argentina; 3 Departamento de Bioquímica Médica y Biología Molecular, Hospital Universitario Virgen Macarena. Facultad de Medicina. Universidad de Sevilla. Avenida Sánchez Pizjuán 4 (41009), Sevilla, Spain; 4 Hospital Nacional Profesor Alejandro Posadas, Buenos Aires, Argentina; 5 Servicio de Ginecología y Obstetricia, Hospital Universitario Virgen Macarena, Sevilla, Spain

**Keywords:** Cancer, Cell biology, Molecular biology, Stem cells

## Abstract

The placental stem cells have called the focus of attention for their therapeutic potential to treat different diseases, including cancer. There is plenty evidence about the antiproliferative, antiangiogenic and proapoptotic properties of the amniotic membrane. Liver cancer is the fifth cause of cancer in the world, with a poor prognosis and survival. Alternative treatments to radio- or chemotherapy have been searched. In this work we aimed to study the antiproliferative properties of the human amniotic membrane conditioned medium (AM-CM) in hepatocarcinoma cells. In addition, we have analyzed the regulation of pro and antiOncomiRs expression involved in hepatocarcinoma physiology. We have determined by ^3^H-thymidine incorporation assay that AM-CM inhibits DNA synthesis in HepG2 cells after 72 h of treatment. AM-CM pure or diluted at 50% and 25% also diminished HepG2 and HuH-7 cells viability and cell number. Furthermore, AM-CM induced cell cycle arrest in G2/M. When proliferation mechanisms were analyzed we found that AM-CM reduced the expression of both Cyclin D1 mRNA and protein. Nuclear expression of Ki-67 was also reduced. We observed that this CM was able to promote the expression of p53 and p21 mRNA and proteins, leading to cell growth arrest. Moreover, AM-CM induced an increase in nuclear p21 localization, observed by immunofluorescence. As p53 levels were increased, Mdm-2 expression was downregulated. Interestingly, HepG2 and HuH-7 cells treatment with AM-CM during 24 and 72 h produced an upregulation of antiOncomiRs 15a and 210, and a downregulation of proOncomiRs 206 and 145. We provide new evidence about the promising novel applications of human amniotic membrane in liver cancer.

## Introduction

Human amniotic membrane (hAM) is well known for many of its beneficial properties and its promising applications. The amnion is a membranous sac that contains the fetus and the amniotic fluid, and is composed of three major layers of a single epithelial layer, a basement membrane and an avascular mesenchyme^1^. Amniotic membrane includes cells with a number of highly attractive features for cellular therapies, with stemness characteristics and the known advantages of the placental tissues for regenerative medicine^2^. Placenta and fetal membranes are obtained easily without the need of invasive techniques, there is an unlimited availability, their use is not associated with ethical or legal impediments, they are not tumorigenic and they display immunomodulatory properties^3–5^. In addition to the promising potential of amniotic cells in the regenerative medicine field^6–8^, hAM has been largely used in medical treatments since it is able to promote re-epithelization, to decrease inflammation and fibrosis, and to inhibit angiogenesis, among others properties^9^. Amniotic membrane is employed in treatments of different ocular diseases and in the reconstruction of damaged tissue^10^. Recently several authors have reported *in vitro* and *in vivo* results that support the theory that amniotic membrane could possess antitumoral properties^11^. Seo *et al*. have hypothesized about these properties, based on its antiangiogenic, immunoregulatory and proapoptotic activities^12^. Amniotic epithelial (hAECs) or mesenchymal cells (hAMCS) secrete a large amount of antiangiogenic factors like interleukin-1 receptor antagonist, collagen XVIII, IL-10, thrombospondin-1 and tissue inhibitor of metalloprotease (TIMP-1, -2, -3, and -4)^13^. As well, they have been described to express the pigment epithelium-derived factor (PEDF), which is known to inhibit proliferation of endothelial cells, contributing to hAM antiangiogenic activity^14^. Some authors demonstrated that proliferation of hematopoietic and no hematopoietic cancer cell lines was significantly reduced by co-cultures with hAMCs^15^. Other study has demonstrated that hAM-conditioned medium or hAECs supernatant inhibits proliferation of Hela and breast cancer cells^16^. Magatti *et al*. have reported that mesenchymal stem cells derived from human amniotic membrane significantly inhibit lymphocyte proliferation, suppress the maturation of dendritic cells and eliminate the production of inflammatory cytokines^17^. In addition, the same group has shown that the mesenchymal cells isolated from amniotic membrane exert an antiproliferative effect on cancer cells by inhibiting cell cycle^15^. Other study^16^ showed an increase in caspase-8 and 3 expression in HeLa and breast cancer cells when they are treated with amniotic epithelial cells conditioned medium. This study proposed the amniotic epithelial cells as a better option than the whole hAM because they have antiangiogenesis and antitumoral effects and they could be simply transferable to human body. Nevertheless, it has been demonstrated that there are no appreciable differences between the conditioned medium (CM) derived from the whole membrane and the CM derived from the cells isolated from membrane, regarding lymphocyte proliferation inhibition^18^. These authors suggested that hAM could be an attractive source of soluble factors without the need of complicated cell preparations.

Liver cancer is the fifth most common cause of cancer in the world and hepatocellular carcinoma (HCC) in particular accounts for 90% of liver primary malignancies^19^. HCC is a progressive and complex process usually detected in late stages of the disease and therefore it has poor prognosis and limited survival. The available treatments in case of early detection are liver transplantation, hepatectomy, and chemo- or radiotherapy, if the former are not possible. However, these therapies often fail because of the HCC radio- or chemoresistance^20^. Given the lack of effective therapies, there is an urgency to find alternative treatments to reduce HCC mortality. The knowledge about the mechanisms and the signaling that govern cell proliferation, apoptosis, migration and other processes linked to tumor development is a central issue to understand the bases of the disease and to discover innovative treatments. The main mechanisms responsible for HCC development –cirrhosis and metabolic disorders- have been associated with abnormalities in the critical involved pathways. Targeting signaling cascades like MAPK, PI3K/Akt/mTOR, Wnt/b-catenin, epidermal growth factor (EGF), insulin-like growth factor (IGF), AMP-Activated Protein Kinase (AMPK), among the most important, may help to prevent, delay or revert hepatocarcinogenesis^21^. Despite being one of the most frequent supplements for eukaryotic cell culture media, fetal bovine serum (FBS) contains a complex natural composition almost undefined and variable between lots^22^. In this way, in order to ensure more reproducible experimental conditions, and diminish analytical interfering, serum is commonly removed^23–25^.

Intracellular pathways are extensively affected by serum starvation and the impact not only is variable but also depends on the cell type^26,27^. Cancer cells that are under serum starvation *in vitro* partially imitate metabolically stressed cells *in vivo*, which are trying to adjust to a changed environment. Thus, by inducing certain signals transduction and gene expression, the tumor keeps growing^28^. Although several studies demonstrated the response of different signaling pathways to serum free conditions^29–31^, the results should be carefully analyzed in each case.

A few studies have demonstrated amniotic membrane anticancer effects on HCC. It has been shown that protein extracts derived from processed hAMs (hAMPEs) inhibit metabolic activity of hepatocarcinoma cell lines HepG2 and Hep3B2.1-7^32^. Studies *in vivo* revealed that hAMPEs were able to promote a complete tumor regression in mice inoculated with HepG2 but not with HuH-7 cells. These authors also demonstrated that hAMPEs negatively regulate protein and DNA content in all HCC lines^33^. They showed that these protein extracts have no effect on metabolic activity inhibition or in protein and DNA content on non-tumorigenic cell line^34^. Mamede *et al*. concluded that hAMPE was capable of induce cell death through intrinsic or extrinsic pathways, depending on the treated hepatocarcinoma cell line^33^.

Certain microRNAs (miRs) are increased in tumors and are considered as oncogenes. They usually promote tumor growth by controlling tumor suppression or cell differentiation. Diverse miRs have been found to be significantly overexpressed in different cancers, and in particular, the dissimilar expression of miRs in hepatoma and normal liver cells could reveal a diagnosis of HCC^35,36^. For clinical treatment, the finding of a supplemental strategy to downregulate protumoral miRs expression would fit. To our knowledge, no study has been developed regarding the regulation of HCC miRs by AM-CM.

Despite the diversity of studies about the antitumoral effects of the amniotic membrane and its cells, a limited number has investigated such effects on HCC models and none of them have analyzed the ability of the entire amniotic membrane supernatant to inhibit hepatocarcinoma cells progression. The aim of this work was to analyze the antitumoral properties of the human amniotic membrane conditioned medium (AM-CM) on hepatocarcinoma HepG2 and HuH-7 cells. We also aimed to study the regulation of certain anti- and protumoral miRs expression in hepatocarcinoma cells after AM-CM treatment.

## Results

### Amniotic membrane conditioned medium decreases DNA synthesis and survival of HepG2 cells

**Figure 1:**
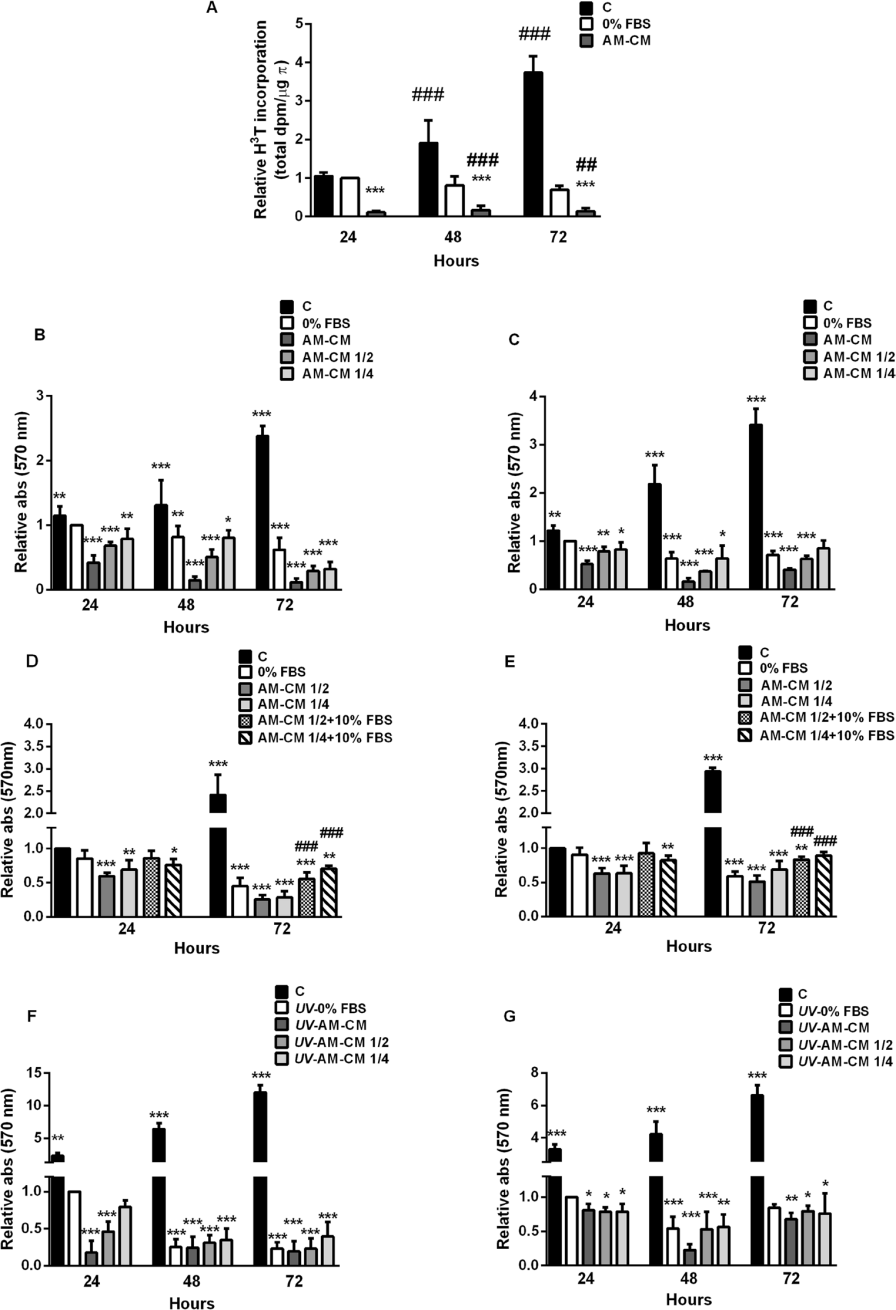
AM-CM inhibits hepatocarcinoma cells proliferation and viability. (**A**) After HepG2 cells treatment with DMEM 10% FBS (C), DMEM 0% FBS (0% FBS) or AM-CM pure, cell lysates were prepared and ^3^H-thymidine incorporation was determined as described in Materials and Methods. HepG2 cells (**B**,**D**) and HuH-7 cells (**C**,**E**) were seeded in 24-wells plate in complete DMEM medium supplemented with 10% FBS (C) or in DMEM 0% FBS (0% FBS) or in AM-CM pure (AM-CM), diluted at 50% (AM-CM 1/2) or diluted at 25% (AM-CM 1/4); or with AM-CM 1/2 10% FBS or AM-CM 1/4 10% FBS (**D**,**E**). Cells viability was determined by MTT test at 24, 48 and 72 h of culture. HepG2 cells (**F**) or HuH-7 cells (**G**) were seeded in 24-wells plate and before AM-CM treatment were exposed to 40 J/m^2^ of UVC (5 sec). After exposure, cells were grown in DMEM-F12 10% FBS (C), DMEM-F12 0% FBS (*UV*-0% FBS) or treated with AM-CM pure (*UV*-AM-CM), 50% diluted (*UV*-AM-CM 1/2) or 25% diluted (*UV*-AM-CM 1/4). Cell viability was determined by MTT test at indicated times. In control cells with DMEM-F12 10% FBS, UV treatment was omitted. Independent experiments were performed in duplicates five times. Results are expressed as means ± S.D. *p < 0.05, **p < 0.01, ***p < 0.001 vs. 24 h serum deprived control. ^##^p < 0.01, ^###^p < 0.001 vs. respective 0% FBS control (**A**,**B**,**C**,**F**,**G**). *p < 0.05, **p < 0.01, ***p < 0.001 vs. 24 h 10% FBS. ^###^p < 0.001 vs. respective 10% FBS control (**D**,**E**)

The deregulation of proliferation and survival is a key process during tumor development. In order to evaluate the AM-CM effect on hepatocarcinoma cells proliferation, we performed [H^3^]-thymidine incorporation assay. Treatment with AM-CM caused a significant decrease in DNA synthesis after 24, 48 or 72 hours, up to 8,7-fold, 5,8-fold and 7-fold, respectively, compared with 0% FBS treatment (Fig. 1A). Moreover, serum deprivation did not significantly inhibit DNA synthesis during the assayed times while AM-CM did. Likewise, HepG2 cells viability reaches a significant 8,6-fold reduction, after 72 h of treatment with AM-CM pure. Both with 50% AM-CM and 25% AM-CM cell viability diminished during all treatments (Fig. 1B). Viability increased in control cells comparing with serum deprived control. Similar results were obtained when we assayed AM-CM on HuH-7, another representative human hepatocarcinoma cell line. Despite AM-CM produced a significant decrease in HuH-7 cell viability, it was minor than the obtained for HepG2 cells (Fig. 1C). In order to exclude serum deprivation effects and since most of the potentially antitumor drugs are studied including serum in media culture, we have assayed survival on HepG2 and HuH-7 cells treated with AM-CM in complete DMEM-F12 10% FBS (Fig. 1D,E).

As seen in Fig. 1D, when HepG2 cells were normal feeded (10% FBS) and treated with AM-CM, viability was significantly diminished up to 4,5-fold after 72 h of treatment, compared with 10% FBS control. This effect was 2,25-fold higher than the effect of AM-CM without serum vs 0% FBS. Similar results were obtained when we analyzed HuH-7 cells (Fig. 1E). HuH-7 cells treated with AM-CM 10% FBS significantly decreased their viability up to 3,4-fold after 72 h of treatment compared with 10% FBS control. AM-CM without serum produced only a 1,4-fold reduction in viability compared with 0% FBS control. These results validate an eventual *in vivo* treatment since AM-CM will successfully act in a tumor environment surrounded by serum.

In order to determine the effect of AM-CM against a more severe cellular damage than serum deprivation, we performed a pretreatment with a pulse of UV radiation. UV treatment prior to serum deprivation induced a higher reduction in HepG2 cell survival at 72 h of treatment comparing with serum deprivation alone (Fig. 1E). Moreover, when cells were treated with AM-CM, viability diminished up to 5,2-fold, after 72 h of treatment. HuH-7 cell survival was also downregulated by AM-CM, after UV treatment, reaching a 4,3-fold reduction after 48 hours of treatment with CM pure (Fig. 1F).

We have also assayed cell proliferation by cell counting. As seen in Supplementary Fig. 1A, AM-CM significantly reduced HepG2 cells number up to 4,9-fold at 72 h of treatment, compared with 24 h 0% FBS. HuH-7 cells were less responsive to treatment, reaching a 1,9-fold reduction in cell number under the same conditions.

It is known that HuH-7 and HepG2 cells have different genetic backgrounds that may result in diverse responses to anticancer treatments^37^. In particular, HepG2 cells express normal p53 and HuH-7 cells express a mutated form. Since we observed that in all cases, HuH-7 cells were less sensitive to the AM-CM treatment, we explored the role of p53, a central regulator of cell proliferation and apoptosis, in this effect. To this end, we measured the viability of Hep3B cells, a liver cell line that lacks p53 expression^38^, by MTT assay. Results shown in Supplementary Fig. 2A demonstrate that Hep3B viability is not significant altered by AM-CM treatment. Moreover, when we evaluated AM-CM effect on other non-liver cell lines we also observed unchanged cell viability. A375 melanoma cell line (Suppl. Fig. 2B), BeWo choriocarcinoma cell line (Suppl. Fig. 2C) and MCF-7 breast cancer cell line (Suppl. Fig. 2D) were not sensible to AM-CM incubation. In particular, MCF-7 cells seem to be the more resistant. Thus, antitumoral effects of AM-CM would be specific for hepatocarcinoma cells.

In conclusion, AM-CM reduced not only proliferation but also survival of hepatocarcinoma cells, causing a major effect in HepG2 than in HuH-7 cells.

### AM-CM arrests hepatocarcinoma cells cycle progression

**Figure 2:**
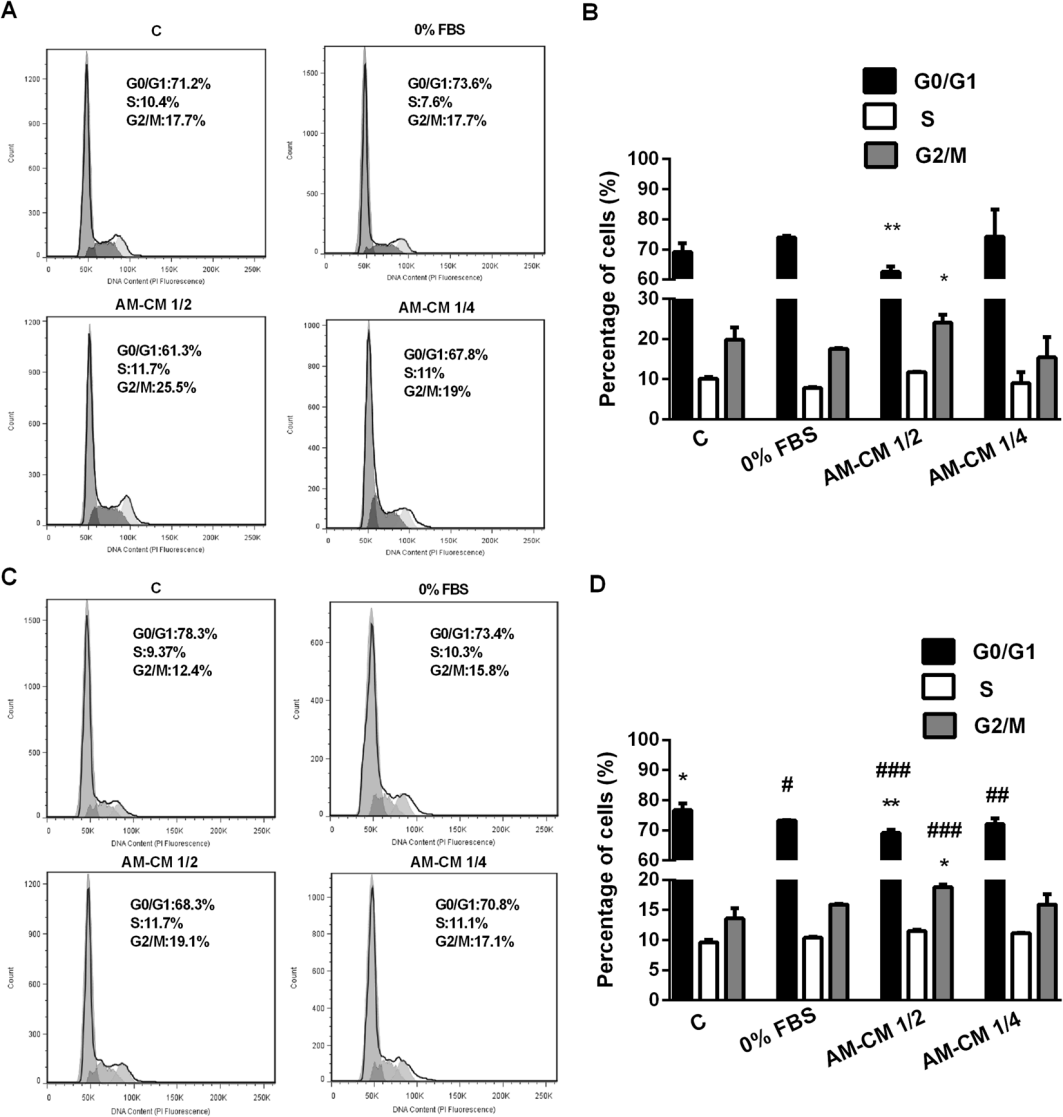
HepG2 cells cycle progression is inhibited by AM-CM treatment. HepG2 cells (5 × 10^5^) were grown in 6-wells plate in DMEM-F12 10% FBS during 24 h. Then, medium was replaced by treatment with DMEM 10% FBS (C), DMEM 0% FBS (0% FBS), AM-CM 1/2 or AM-CM 1/4. After 24 h and 72 h cells were processed and analyzed by flow cytometry, as indicated in Materials and Methods. In (**A**,**C**) the cell population was selected on the basis of size (FSC-H) and granularity (SSC-H) and this population was plotted in PI-A vs PI-W plot in order to discriminate doublet population. Then singlet population was selected and plotted in count vs PI (propidium iodide) intensity graph. Graphs represent the distribution of HepG2 cells in different phases of cell cycle at 24 h (**A**) or 72 h (**C**) of treatment. The peak on the left in all graphs represents the G1 population and peak on the right represents the G2/M population. The valley between these two peaks represents the S phase population (**B**–**D**). The bar diagram represented the % of cells in different phases of cell cycle after 24 h (**B**) or 72 h (**D**) of treatment. Results are expressed as means ± S.D. (n = 3) and one from a representative experiment is shown. *p < 0.05, **p < 0.01 vs. 0% FBS; ^#^p < 0.05, ^##^p < 0.01, ^###^p < 0.01 vs. 10% FBS

Since we observed an inhibition in proliferation and survival of HepG2 and HuH-7 cells treated with AM-CM, we decided to investigate the molecular mechanisms involved in such effect. Thus, we next evaluated cell distribution during the different stages of cell cycle after AM-CM treatment. We quantified DNA by IP staining and cell cytometry analysis. As seen in the histograms displayed in Fig. 2A,C, control HepG2 cells (10% FBS) show a distribution where the left peak corresponds to cells in G1 stage and the right peak to G2/M stage. Our results indicate that 23% of cells were arrested in G2/M phase of the cell cycle after 24 h of treatment with AM-CM 1/2, while cells in G1 significantly diminished from 73% to 63% (0% FBS vs AM-CM 1/2) (Fig. 2B). Following 72 h incubation with AM-CM 1/2 and 1/4, 18% and 17% of cells were arrested in G2/M, respectively. In addition, the percentage of cells in G1 was significantly reduced from 73% to 68% (0% FBS vs AM-CM 1/2) (Fig. 2D).

We also performed cell cycle analysis in HuH-7 cells. We did not observe any change after 24 h of treatment (Suppl. Fig. 3A,B). However, following 72 h incubation with AM-CM 1/2 and 1/4, 21% and 23% of cells were significantly arrested in G2/M, respectively. Moreover, cells in G1 diminished from 77% (0% FBS) to 67% (AM-CM 1/4) (Suppl. Fig. 3C,D). Taken together, these results indicate that AM-CM is preventing the progression of hepatocarcinoma cells from G2/M to G1.

### AM-CM downregulates key regulatory proliferation proteins expression

**Figure 3:**
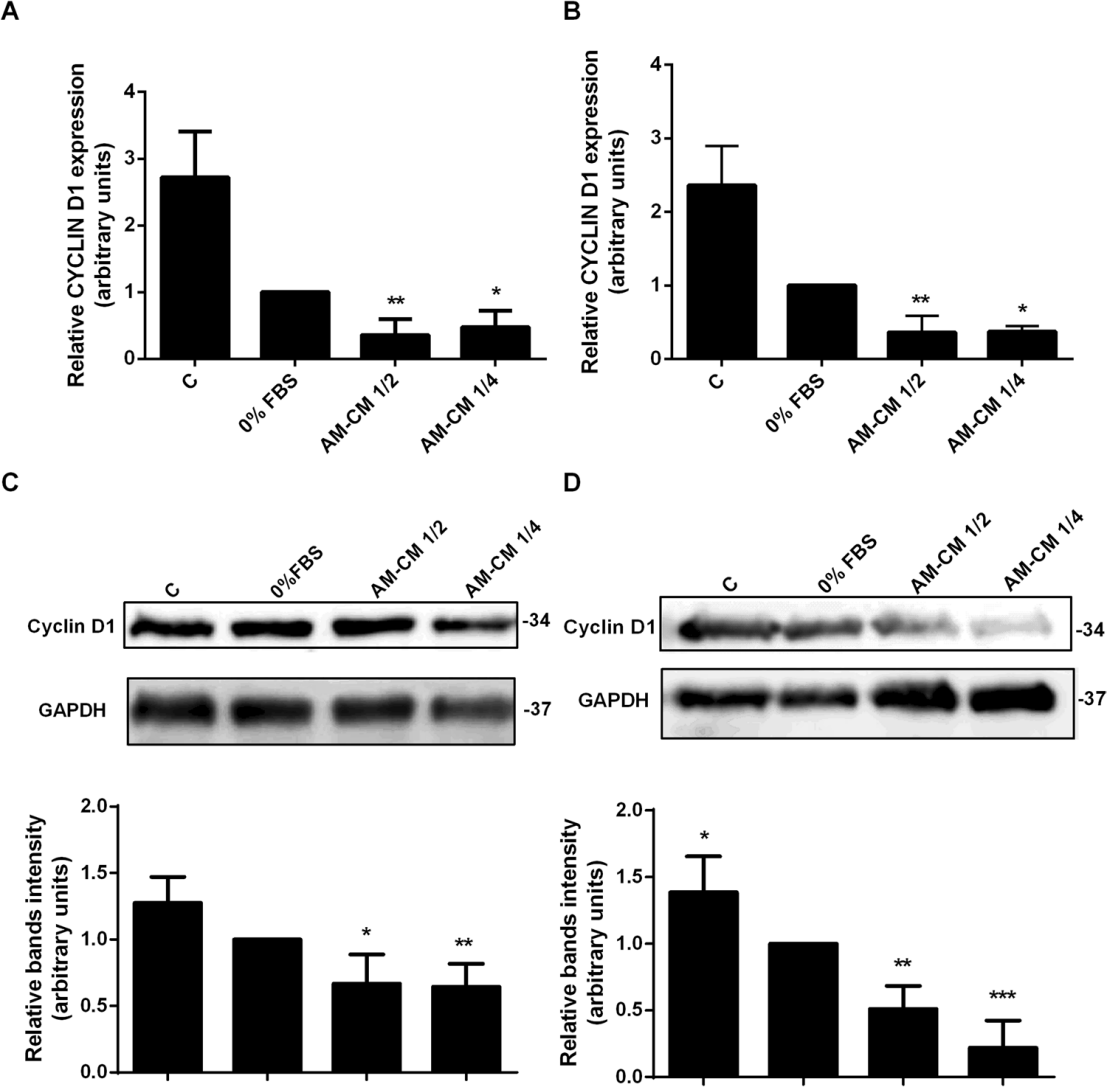
Key proliferation proteins expression is downregulated by AM-CM treatment. HepG2 cells were incubated with DMEM-F12 10% FBS (C), DMEM-F12 0% FBS, AM-CM 50% diluted (AM-CM 1/2), AM-CM 25% diluted (AM-CM 1/4) during 24 (**A**) or 72 h (**B**) before RNA extraction. CYCLIN D1 mRNA was measured by quantitative real time PCR. CYCLOPHILIN and GAPDH were used as internal standards. (**C**,**D**) HepG2 cells were seeded in 10-cm plate and incubated with complete DMEM-F12 medium supplemented with 10% FBS (C), or without serum (0% FBS), or with AM-CM 50% (AM-CM 1/2) or AM-CM 25% (AM-CM 1/4). After 24 h (**C**) or 72 h (**D**). Cyclin D1 expression in cell extracts was determined by Western blot. Molecular weights were estimated using standard protein markers. Loading controls were performed by immunoblotting the same membranes with anti-GAPDH. Lower panels show bands densitometry. Full-length blots are available in Supplementary Dataset. Molecular weight (kDa) is indicated at the right of the blot. *p < 0.05, **p < 0.01, ***p < 0.01 vs. 0% FBS.

Since proliferation is associated to progression in cell cycle, and we observed an inhibition in cell cycle progression after AM-CM treatment, we prompted to analyze which components of the cell cycle machinery might be involved in these effects. Thus, we first evaluated the expression of Cyclin D1 in HepG2 cells treated with AM-CM 1/2 and AM-CM 1/4. Figures 3A,B show AM-CM 1/2 induced a downregulation in CYCLIN D1 mRNA expression, by 2,8-fold, after 24 and 72 h of treatment, compared with serum deprived control. Similar results were obtained when Cyclin D1 protein expression was evaluated by Western blot. We observed that AM-CM produced a significant decrease in Cyclin D1 expression, with a maximal of 4,8-fold reduction after 72 h of treatment with AM-CM 1/4 (Fig. 3C,D). We have also examined Cyclin D1 expression after AM-CM treatment in HuH-7 cells. As seen in Supplementary Fig. 4A,B, AM-CM caused a significant downregulation in CYCLIN D1 mRNA expression, reaching a maximal of 3,3-fold reduction with AM-CM 1/2, at 72 h of treatment. Similar results were obtained when we analyzed Cyclin D1 expression by Western blot, where this protein reduced its level up to 3,3-fold following 72 h of AM-CM 1/4 treatment.

Since Ki-67 binds the perichromosomal layer only in actively growing and dividing cells, it is extensively used as a proliferation marker.

**Figure 4:**
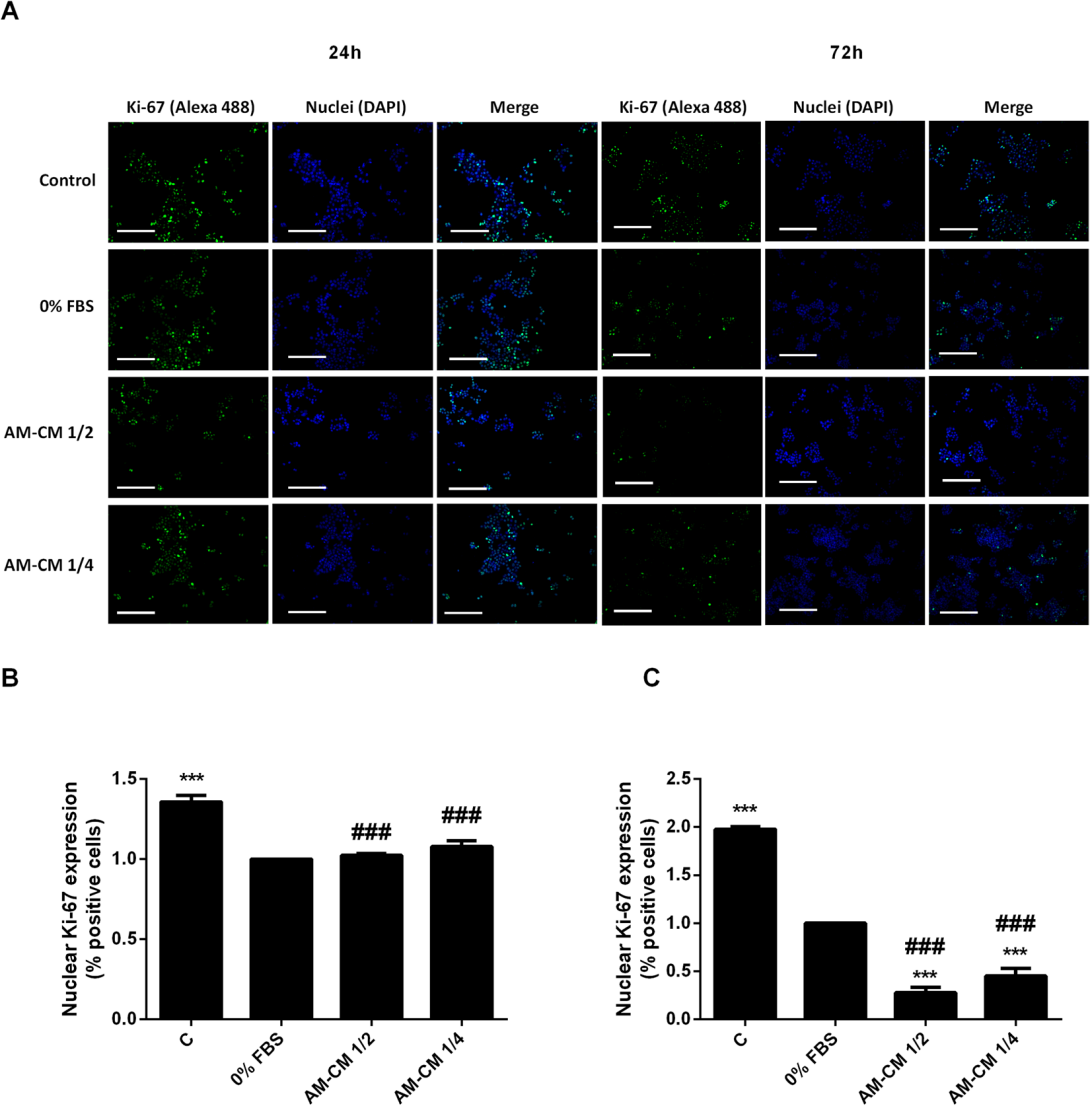
Ki-67 expression is diminished after AM-CM treatment. (**A**) HepG2 cells were seeded in 24-wells plate and treated with DMEM-F12 10% FBS (C), DMEM-F12 0% FBS (0% FBS), AM-CM 50% diluted (AM-CM 1/2), AM-CM 25% diluted (AM-CM 1/4), at indicated times. Cells were fixed and Ki-67 expression (green) was detected using Alexa-488 conjugated secondary antibody, at indicated times. Representative micrographs from HepG2 taken at 10X are shown. The nuclei were stained with DAPI (blue). (**B**,**C**) Graph shows the number of HepG2 nucleus positive for Ki-67 at 24 h (**B**) and 72 h (**C**) of treatment. Scale Bar: 100 μm. Results are expressed as means ± S.D. (n = 5) and one from a representative experiment is shown. ***p < 0.001 vs. 0% FBS; ^###^p < 0.001 vs. 10% FBS.

Thus, we decided to analyze Ki-67 expression in HepG2 cells treated with AM- CM during 24, 48 and 72 h (Fig. 4). We observed by immunofluorescence that Ki-67 expression was significantly diminished with AM-CM 50% and 25% by 5,5-fold and 3,4-fold respectively, after 72 h of treatment, comparing with 0% FBS control (24 h). All treatments caused a significant decrease when comparing with 10% FBS control. Therefore, AM-CM is inhibiting cell cycle progression, viability and regulatory proliferation proteins expression, especially after 72 h of treatment, which would indicate a regression in hepatocarcinoma cells growth.

### P53 expression increases after AM-CM treatment

**Figure 5:**
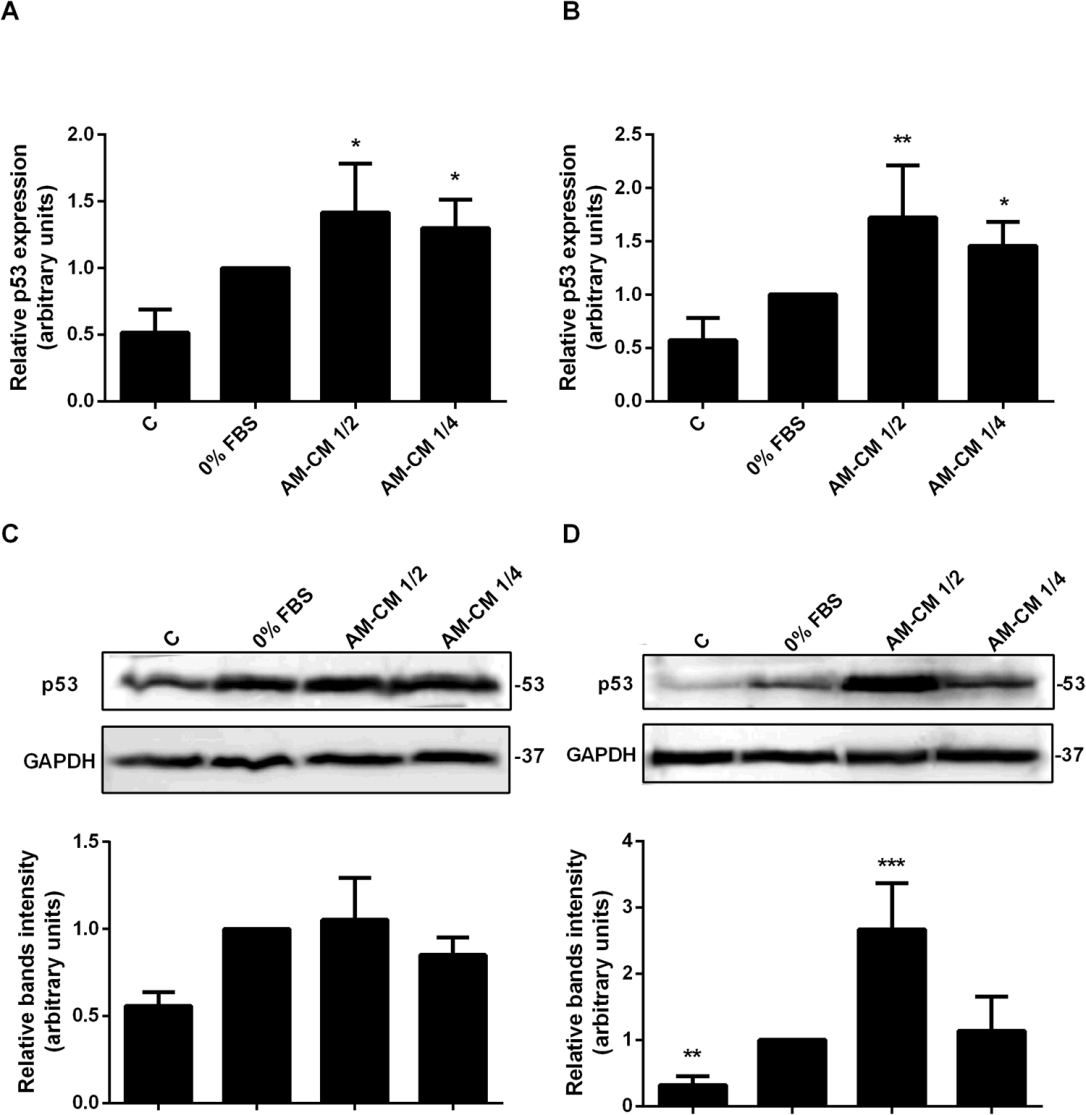
P53 expression increases in HepG2 cells after AM-CM treatment. HepG2 cells were plated in complete DMEM-F12 medium 10% FBS (C), in DMEM-F12 0% FBS (0% FBS), in AM-CM diluted at 50% (AM-CM 1/2) or at 25% (AM-CM 1/4) and incubated during 24 h (**A**) or 72 h (**B**) before total RNA extraction. P53 mRNA was measured by quantitative real time PCR. CYCLOPHILIN and GAPDH were used as internal standards. (**C**,**D**) HepG2 cells were seeded in 10-cm plate and incubated with complete DMEM-F12 10% FBS (C), or without serum (0% FBS), or with AM-CM 50% (AM-CM 1/2) or AM-CM 25% (AM-CM 1/4). Cell extracts were prepared at indicated times and proteins were separated on SDS-PAGE gels. P53 expression at 24 h (**C**) or 72 h after treatment (**D**) was determined by Western blot. Molecular weight was estimated using standard protein markers and is indicated at the right of the blot. Loading controls were performed by GAPDH detection. Bands densitometry is shown in lower panels. Full-length blots are available in Supplementary Dataset. Results from a representative experiment are shown and expressed as means ± S.D. for five independent experiments performed in duplicates. For Western blot, representative results from three replicates are shown. *p < 0.05, **p < 0.01, ***p < 0.001 vs. 0% FBS.

The tumor suppressor protein, p53, participates in transactivation of downstream target genes that leads to cell cycle arrest, senescence, and apoptosis, preventing the proliferation and existence of damaged cells. In this context, we evaluated the expression of the proapoptotic protein p53 in HepG2 cells treated during 24 or 72 h with AM-CM diluted to the half and to the forth. Results are shown in Fig. 5. P53 mRNA expression significantly augmented up to 1,7-fold with AM-CM 1/2 treatment, comparing with control, after 72 h in culture. AM-CM 1/4 treatment also caused a significant increase in p53 expression (Fig. 5A,B). Comparable results were observed by Western blot assays. AM-CM significantly upregulated p53 expression when it was added half diluted -2,7-fold- after 72 hours of treatment (Fig. 5D). Similar results were observed in HuH-7 cells. P53 mRNA as well as p53 protein expression significant increased their levels after AM-CM treatment (Supplementary Fig. 5A–D).

**Figure 6:**
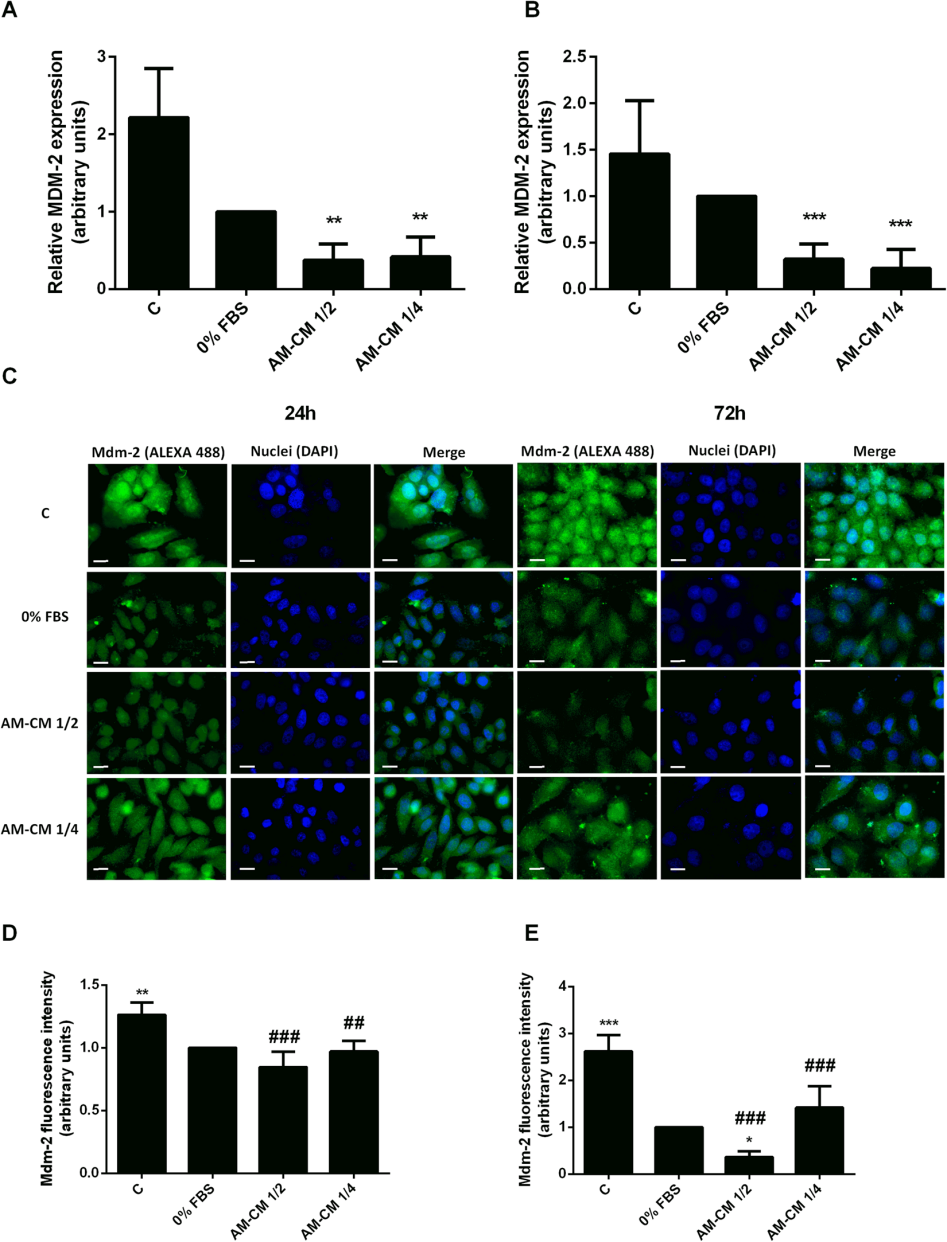
Mdm-2 expression decreases in HepG2 cells after AM-CM treatment. HepG2 cells were plated in DMEM-F12 medium supplemented with 10% FBS (C), in DMEM-F12 0% FBS (0% FBS), in AM-CM diluted at 50% (AM-CM 1/2), AM-CM at 25% (AM-CM 1/4) and incubated during 24 h (**A**) or 72 h (**B**) before RNA extraction. MDM-2 mRNA expression was measured by quantitative real time PCR. CYCLOPHILIN and GAPDH were used as internal standards. (**C**–**E**) HepG2 cells incubated in DMEM-F12 10% FBS (C), DMEM-F12 0% FBS, AM-CM 50% dilution (AM-CM 1/2), or AM-CM 25% dilution (AM-CM 1/4), during indicated times. Cells were fixed at specified times and Mdm-2 expression (green) was detected using Alexa-488 conjugated secondary antibody. Representative micrographs at 60X from HepG2 cells at 24 h (**C**) and 72 h (**D**) are shown. The nuclei were stained with DAPI (blue). Graphs below show Mdm-2 fluorescence intensity in HepG2 cells at 24 h (**E**) and 72 h (**F**) of treatment. Scale Bar: 8 μm. Results are expressed as mean ± S.D. (n = 5). **p < 0.01, ***p < 0.001 vs. 0% FBS; ^##^p < 0.05, ^###^p < 0.001 vs 10% FBS (C).

The oncoprotein and E3 ubiquitin ligase, Mdm-2 negatively regulates both p53 stability and activity. Mdm-2 not only promotes p53’s translocation to the cytoplasm from the nucleus blocking transcription of p53 targets but also induces p53 proteasome-mediated degradation through its ubiquitination^39^. In this way, we have determined Mdm-2 expression in HepG2 cells treated with AM-CM, both by *q*RT-PCR (Fig. 6A,B) and immunofluorescence (Fig. 6C–E). As expected, conditioned medium produced a downregulation of MDM-2 mRNA expression both at 24 and 72 h of treatment, triggering a maximum of 3,1-fold decrease with 50% CM dilution after 72 h. On the other hand, immunofluorescence images revealed that Mdm-2 protein expression also diminished to a maximum of 2,7-fold when AM-CM 1/2 treatment was applied during 72 h. In all cases, Mdm-2 expression was significant downregulated comparing with 10% FBS control. Taking together these results demonstrate that unknown factors released by the amniotic membrane to the environment have the ability to inhibit the cell cycle progress of tumoral cells by regulating key related proteins.

### AM-CM upregulates p21 expression and its nuclear localization

**Figure 7:**
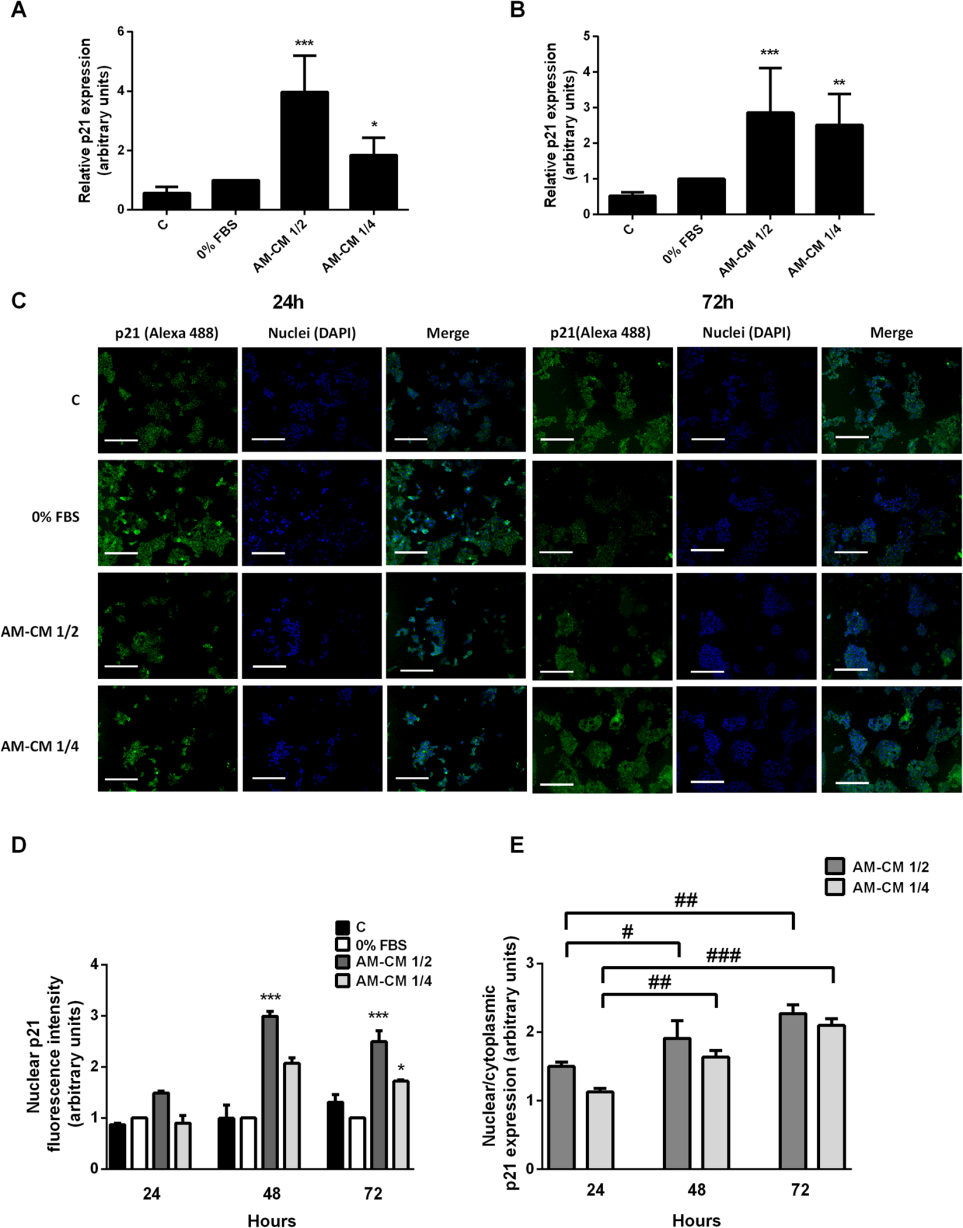
AM-CM induces p21 expression and translocation to HepG2 cells nucleus. HepG2 cells were plated in complete DMEM-F12 medium supplemented with 10% FBS (C), in DMEM-F12 0% FBS (0% FBS), in AM-CM diluted at 50% (AM-CM 1/2), AM-CM at 25% (AM-CM 1/4) and incubated during 24 h (**A**) or 72 h (**B**) before RNA extraction. Total RNA was extracted as described in Materials and Methods. P21 mRNA was measured by quantitative real time PCR. CYCLOPHILIN and GAPDH were used as internal standards. (**C**) HepG2 cells were seeded in 24-wells plate and incubated in DMEM-F12 10% FBS (C), DMEM-F12 0% FBS, AM-CM 50% dilution (AM-CM 1/2), or AM-CM 25% dilution (AM-CM 1/4), during indicated times. P21 expression (green) was detected in fixed cells using Alexa-488 conjugated secondary antibody. Representative micrographs at 10X from HepG2 at 24 h and 72 h are shown. The nuclei were stained with DAPI (blue). (**D**) Graph shows quantification of nuclear p21 fluorescence intensity in HepG2 cells. Scale Bar: 100 μm. (**E**) Graph shows p21 nuclear localization related to cytoplasmic localization after AM-CM 1/2 and AM-CM 1/4 treatments. Results are expressed as mean ± S.D. for five independent experiments. *p < 0.05, **p < 0.01, ***p < 0.001 vs. 0% FBS; ^#^p < 0.05, ^#^p < 0.01, ^###^p < 0.001 vs AM-CM 1/2 or 1/4 24 h treatment.

As we previously shown, AM-CM treatment increments p53 expression in HepG2 and HuH-7 cells. One of the main effectors of p53 responses is the cyclin kinases inhibitor p21, a protein that suppresses cell cycle progression. Thus, we evaluated p21 expression in HepG2 cells treated with AM-CM 1/2 and 1/4 during 24 and 72 h. As seen in Fig. 7A,B, p21 mRNA expression increased after 24 and 72 hours of treatment up to 3,9-fold comparing with serum depleted control. Similarly, p21 expression significantly augmented in HuH-7 cells after 72 h of treatment with AM-CM 1/4 (Suppl. Fig. 5E,F). It is generally accepted that the major role of p21 in cell growth and survival differs according to its subcellular distribution: cytoplasmic concentration of p21 supports cell growth and survival, whereas its nuclear concentration can lead to cell cycle arrest and growth inhibition because of DNA damage^40^. In order to determine p21 localization in hepatocarcinoma cells after treatment with AM-CM, we performed immunofluorescence assays, at 24 h and 72 h of culture. Figures 7C,D show a significant increase in nuclear p21 expression, at 24, 48 and 72 h of treatment, reaching up to 3-fold stimulation with AM-CM 1/2 at 48 h. In Fig. 7E the changes in nuclear/cytoplasmic p21 localization (%) after AM-CM treatment were plotted. The p21 cellular localization changed after AM-CM treatment. P21 is induced to translocate to the nucleus when HepG2 cells are incubated with AM-CM, indicating a proapoptotic and antiproliferative role of p21 induced by AM-CM treatment.

These results reinforce the idea that human AM-CM inhibits proliferation of hepatocarcinoma cells by affecting cell cycle proteins expression and localization. Further investigations will be needed to determine if regulation of apoptosis is also involved in these effects and to unravel the mechanisms by which it occurs.

### Anti- and protumoral microRNAs expression is modulated by AM-CM

**Figure 8:**
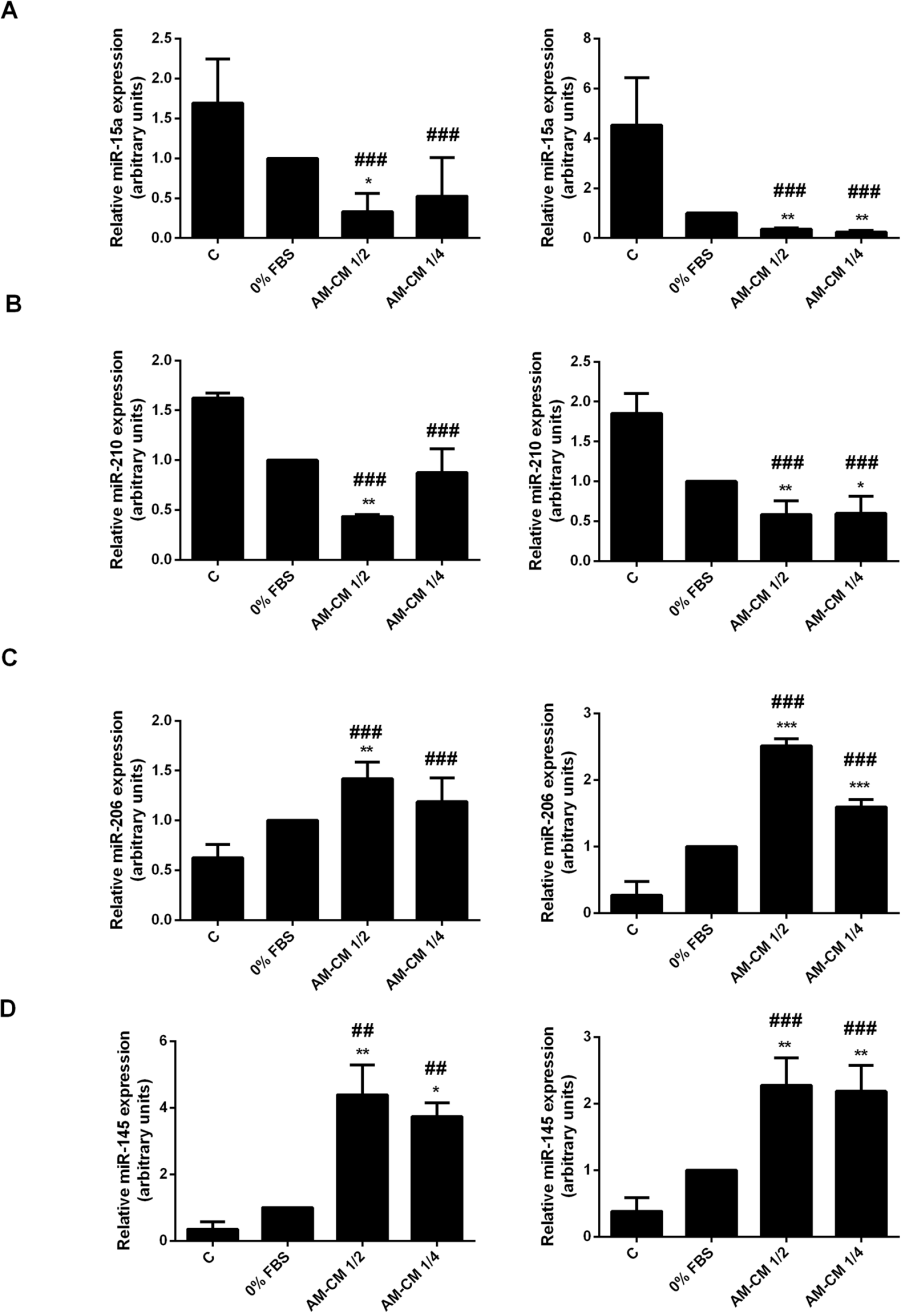
Pro and antitumoral miRs expression is modulated by AM-CM in HepG2 cells. (**A**,**D**) HepG2 cells were plated in complete DMEM-F12 medium 10% FBS (C), in DMEM-F12 0% FBS (0% FBS), in AM-CM diluted at 50% (AM-CM 1/2), AM-CM at 25% (AM-CM 1/4) and incubated during 24 h (left) or 72 h (right) before RNA extraction. Relative expression of miR-15a (**A**), miR-210 (**B**), miR-206 (**C**), miR-145 (**D**) was determined by qRT-PCR. RNU6B was used as internal control for normalization. *p < 0.05, **p < 0.01, ***p < 0.001 vs. 0% FBS; ^#^p < 0.05, ^###^p < 0.001 vs 10% FBS.

MicroRNAs (miRs) have recently been described because of their key roles in physiological and pathological processes. They act by targeting specific genes through translational repression or mRNA degradation. In particular, some studies have identified the roles of miRs in HCC^35,41,42^ in order to obtain a better insight into the pathology. In this context, we have evaluated the changes in miR-15a, miR-210 (pro-oncomiRs), miR-206 and miR-145 (anti-oncomiRs) expression in HepG2 and HuH-7 cells during AM-CM treatment. As seen in Fig. 8A,B, pro-oncomiRs 15a and 210 expression was significantly downregulated by AM-CM, after 24 and 72 h of treatment, reaching a maximal of 5,8-fold reduction for miR-15a and 2,4-fold reduction for miR-210. On the contrary, anti-oncomiRs 206 and 145 were significantly upregulated in HepG2 cells treated with AM-CM during 24 and 72 h. MiR-206 increased up 2,5-fold after 72 h and miR-145 up to 4,3-fold after 24 hours of treatment (Fig. 8C,D).

**Figure 9:**
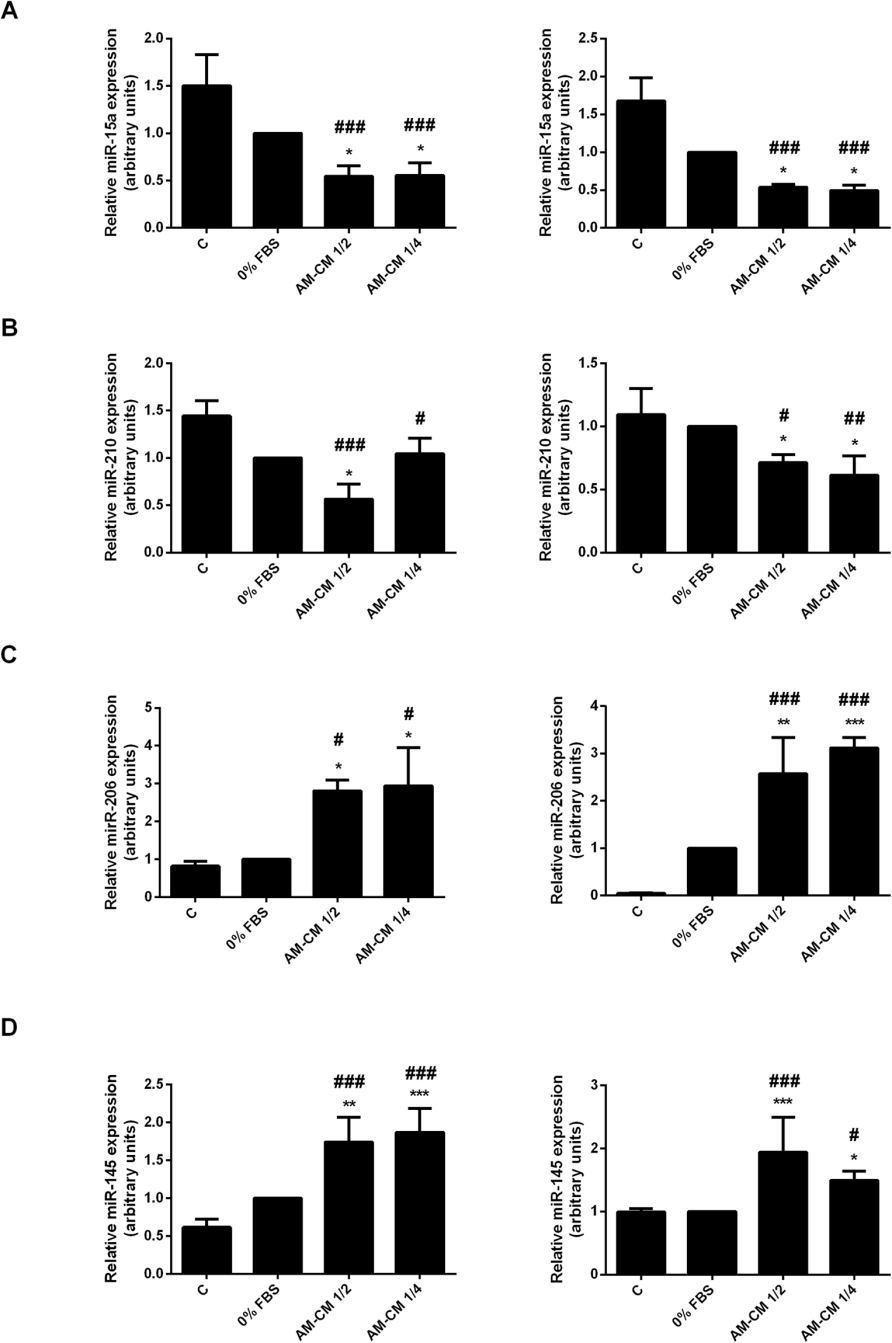
Pro and antitumoral miRs expression is modulated by AM-CM in HuH-7 cells. (**A**–**D**) HuH-7 cells were plated in complete DMEM-F12 medium supplemented with 10% FBS (C), in DMEM-F12 0% FBS (0% FBS), in AM-CM diluted at 50% (AM-CM 1/2), AM-CM at 25% (AM-CM 1/4) and incubated during 24 h (left) or 72 h (right) before RNA extraction. Total RNA was extracted as described in Materials and Methods. Relative expression of miR-15a (**A**), miR-210 (**B**), miR-206 (**C**), miR-145 (**D**) was determined by *q*RT-PCR. RNU6B was used as internal control for normalization. *p < 0.05, **p < 0.01, ***p < 0.001 vs. 0% FBS; ^#^p < 0.05; ^##^p < 0.01, ^###^p < 0.001 vs 10% FBS.

Comparable results were obtained when we analyzed miR-15a, miR-210, miR-206 and miR-145 expression in HuH-7 cells treated with AM-CM. As expected, AM-CM caused a significant decrease of miR-15a and miR-210 in HuH-7 cells (Fig. 9A,B). MiR-15a reduced its expression by 1,9-fold and miR-210 by 1,8-fold, after 24 h of treatment. Both at 24 and at 72 h after treatment, anti-oncomiRs were significantly upregulated, up to 3-fold with 25% dilution (miR-206) and 8,4-fold (miR-145) comparing to serum-free control (Fig. 9C,D).

Taking in account the observed upregulation of oncomiRs and the downregulation of antioncomiRs, support the findings about the antitumoral properties of the amniotic membrane CM on human hepatocarcinoma cells. In summary, the showed results suggest that AM-CM inhibits cell cycle progression, downregulating survival and proliferation of hepatocarcinoma cells.

## Discussion

The finding of effective therapies to fight against cancer has been one of the scientific challenges since a long time ago. In particular, treatments and cures for hepatocarcinoma, the main frequent primary liver cancer worldwide, have been extensively studied. HCC is the fifth most common cancer and the second leading cause of cancer death^43^, representing a global health problem. Despite the multiple treatments available, like liver transplantation, chemo- or radiotherapies, resection, none of them are totally effective and there is a severe lack of appropriate donor organs^44^. In this context, alternative therapies are being investigated and particularly those related with stem cell biology and regenerative medicine.

The human amniotic membrane not only has several functions during the gestational period but also has been described as an important source of stem cells^5^. The amniotic membrane is briefly formed by an epithelial layer, a basement membrane and a mesenchymal layer^45^. The first one is composed by cuboidal amniotic epithelial cells (hAECs). The mesenchymal layer contains cells with high mobility named human amniotic mesenchymal cells (hAMCs). Both types of cells enclose attractive stemness characteristics and are intensively studied for their use in regenerative medicine^8,45–47^. In addition, antitumoral properties have been recently described not only for hAECs and hAMCs but also for the entire human amniotic membrane^9,15,48–50^. Some recent studies have speculated that amniotic cells are capable of inhibit immune cells proliferation^51,52^ and promote apoptosis of B and T lymphocytes^53,54^ by secreting soluble factors like TNF-α or TGF-β. The antiproliferative effect of amniotic membrane was also demonstrated on cancer cells by Magatti *et al*.^15^. They showed that amniotic membrane derived mesenchymal cells arrest the cell cycle of Jurkat and U937 cancer cells in G0/G1 phase. In this way, we have demonstrated that AM-CM significantly inhibits DNA synthesis in hepatocarcinoma HepG2 cells after 24 and 72 h of treatment, compared with serum deprived control. Hepatocarcinoma cell number also decreased after AM-CM treatment. Similar results were obtained when viability was determined. Moreover, the double damage caused by UV irradiation plus serum starvation was lower than that generated by the AM-CM, which caused a significant downregulation of cell survival after 24 h of treatment and on. HuH-7 hepatocarcinoma cells treated with AM-CM threw comparable results.

In the last years the rise of the information about the signaling pathways that control HCC has allowed the identification of potential therapeutic targets and drugs. These pathways may vary from *in vivo* to *in vitro* conditions depending on multiples factors. Serum starvation in particular, deregulates diverse signaling pathways that might affect the expression of cellular proteins^26,27^. Nevertheless, to ensure further reproducible experimental conditions, and reduce analytical interfering, serum is commonly removed^23,24^.

In the present study, we have generated conditioned media from the amniotic membrane without serum inclusion. Fetal bovine serum is not suitable for a safe human clinical use and we aim to develop a possible antitumoral treatment for human hepatocarcinoma. Thus, we have used serum deprived media as control. Even so, during an *in vivo* treatment AM-CM will reach a serum rich environment. Based on this, we have performed control assays, including a control group of cells treated with AM-CM in complete DMEM-F12 10% FBS. We have observed that HepG2 cells treated with AM-CM 10% FBS significantly diminished their viability, compared with 10% FBS control, and this effect was twice as high as that with serum removed. Similar results were obtained with HuH-7 cells. Thus, 10% FBS control validates our previous and following assays, since in a future hypothetic treatment, AM-CM probably will reach a serum rich tumor environment and will effectively exert an antitumor action as well. In this way, Rossi *et al*.^18^ have demonstrated that soluble factors released in CM from cultures of amniotic mesenchymal cells and AM possess the ability to inhibit T lymphocyte proliferation. They prepared serum free CM and when they added FBS to CM as control, they did not observe changes in that inhibitory ability.

While many aspects of the culture in serum-free conditions have been carefully studied many other still remain unknown^27^. Normal cells and cancer cells respond different when serum is eliminate^25^ and signaling pathways are responsible for these reactions. A variety of signals that elicits the HCC has been study and described in a diversity of culture conditions^21^. Central proteins of these pathways as MAPK, PI3K or AMPK, could be potentially use as target therapies in HCC^55–61^. These targets will reply different in presence or absence of serum and therapies need to be design based on this. Further experiments will be required to get a deeper thoughtful about the mechanisms triggered by serum deprivation condition and to bring these conditions closer to *in vivo* situations.

Based in our results, HepG2 cells seem to be more sensible to AM-CM treatment than HuH-7 cells, since viability diminution is noticeably higher in the former. HuH-7 and HepG2 cell lines diverge in their genetic background therefore they have different responses to chemotherapeutic agents. Among the numerous differences between HepG2 and HuH-7 cell lines, some stand out like their methylation status^62^, the lack of the detoxifying enzyme COX2 in HepG2 cells^63^ or the highest expression of hepatocyte differentiating transcription factors CEBPα and HNF4α in HuH-7 cells^64^.

One prominent difference lies in p53 expression, a protein that has a central role in proliferation control and apoptosis induction^37^. HepG2 cells express wild type p53 while HuH-7 cells express a mutated form of p53. Based on it, we speculate that this could be one the reasons why HuH-7 cells are more resistant to AM-CM treatment. Indeed, we observed that Hep3B cells which lack p53 protein expression^38^ were not affected by AM-CM treatment when proliferation was assayed. Nevertheless, since only a general viability assay was performed, further studies will be necessary to confirm the putative role of p53 on AM-CM antitumoral effect.

Di Germanio *et al*. have demonstrated that soluble factors contained in the conditioned medium derived from rat AECs are capable of inhibit the proliferation of HepG2 cells^65^. In the same way it has been shown that protein extracts from amniotic membrane generate an inhibition in metabolic activity of HepG2 (64%) and HuH-7 (25–50%) cells^32^ after 72 h of incubation. In addition these authors observed that HepG2 and Hep3B2.1-7 hepatocarcinoma cells, together with PANC-1 cells, were the most susceptible cell lines to amniotic membrane extracts treatment. In our results, we have shown that proliferation of Hep3B cells is not affected by AM-CM treatment. In this way, it is worth to notice that we are analyzing the effects of all the soluble factors releasing by the amniotic membrane. In their work, Mamede *et al*. aimed to study the action of the membrane protein extracts only^32^. Other bioactive molecules distinct from proteins, like extracellular vesicles^66^ or prostaglandins^18^ could be also exerting key actions. The incubation time used to generate the CM from membrane is also another variable to consider. Thus, the diversity in CM composition generated by the different protocols probably underlines the functional changes in AM-CM effect on hepatocarcinoma cells. Further experiments will be needed to establish its composition in each case. In particular, we have demonstrated that other non-liver cell lines like A375, MCF-7 or BeWo are not responsive to AM-CM treatment, when viability was tested. Thus, AM-CM treatment would specially fit for hepatic tumor cells.

Cell cycle arrest is an important anticancer mechanism. Parolini *et al*. have demonstrated that amniotic mesenchymal cells arrest cell cycle of cancer cells in G0/G1 phase, preventing the progression to S phase and exerting an antiproliferative action probably through the release of unknown soluble factors^15^. Instead, other group established that protein extracts from amniotic membranes induced G2/M phase arrest in HepG2 and HuH-7 cells, accompanied by a huge DNA damage^33^. Actually, our cell cycle analysis revealed that HepG2 and HuH-7 cells treated with AM-CM decrease their cell cycle progression, and the number of cells in G2/M augments. In this work we have observed that AM-CM both at 50% and at 25% caused an arrest in HepG2 and HuH-7 cell cycle at G2/M phase, inducing a decrease in proliferation and therefore leading to an antitumor action.

The levels of Cyclin D1 in G2/M phase of cell cycle are crucial to define to undergo another round of replication. Since Cyclin D1 is upregulated during G2 phase of the cell cycle we examined its expression in hepatocarcinoma cells after AM-CM treatment. We have observed that conditioned medium from amniotic membrane inhibits Cyclin D1 expression in HepG2 and HuH-7 cells, not only at 24 but also at 72 h of treatment. Similarly, Ki-67 expression was also significantly downregulated in HepG2 cells. Since Cyclin D1 promotes cell cycle progression, favoring proliferation, and inhibits cell migration through p27 stabilization, it has been suggested its role in different types of cancer^67,68^. However, overexpression of Cyclin D1 in common cancers is believed to be a consequence of defective regulation at a posttranslational level^69^. In HCC in particular, associations between Cyclin D1 and cell activities inhibition remains unclear. It has been reported that Cyclin D1 expression in HepG2 cells is higher than in normal L02 hepatic cell line. Moreover, Cyclin D1 expression was higher in HCC than in normal hepatic tissue. In addition, inhibition of Cyclin D1 mRNA caused an increase in Caspase-3 activity and a downregulation in Bcl-2 and c-myc expression in HepG2 cells, thus promoting apoptosis and preventing proliferation^70^. Since Cyclin D1 overexpression is usually a result of genetic mutation, elevated Cyclin D1 expression is a consequence of cancer formation. In this way, the reduction of Cyclin D1 mRNA and protein expression in hepatocarcinoma cells through AM-CM treatment, would probably reduce tumor growth. Similarly, Ki-67 is generally used as a proliferative and prognostic marker in HCC and different associations between Ki-67 and HCC physiopathology have been investigated^71–74^. In fact, Ki-67 expression was shown to be directly related to hepatic tumor growth rate^75^. In addition, Ki-67 is expressed in G1, S and G2/M phases during cell cycle but not in G0^76^. Our results showed that AM-CM is able to reduce Ki-67 expression.

During HCC development, different alterations in the expression of three key proteins involved in cell cycle regulation occur: p53, p21 and Mdm-2. In the wild type form, p53 acts as an oncosuppresor, either by initiating cell cycle arrest or apoptosis. Deregulation or mutations in p53 gene expression cause a resistance to apoptosis. In this way, p21/WAF1 protein, which inhibits cell cycle progression and cell growth, cannot be activated by p53^77^. Mdm-2 protein binds p53 and negatively regulates its stability and activity. Mdm-2 binds to p53 blocking the transcription of target promoters and stimulating proteasome degradation through ubiquitination. At the same time, p53 induces Mdm-2 transcription creating a negative feedback loop^78^. In particular, in HCC there are alterations in p53-Mdm-2 and in p53-p21 pathways^79^. These alterations include mutations in the DNA binding domain of p53 which lead not only to a minor affinity to the sequence-specific response elements of its target genes, but also to a decrease of p53 mediated expression of Mdm-2^80^. In tumorigenic cell growth, key sites in Mdm-2 and p53 are not phosphorylated, conducting to an increase in Mdm-2 expression and an inhibition of p53 activity, which derives in cell cycle checkpoint control evasion^81^. In addition, it is known that p21 expression augments proportionally to the suppression of genes that are central for cell cycle progression and to the induction of those associated to senescence^82^. There are controversy about the role and abundance of p21 in human HCC. It is believed that p21 expression is related to tumorigenesis since its expression is significantly higher in normal liver that in HCC tissues. However, Zhang *et al*.^77^ have reported an overexpression of p21 in HCC tissues probably due to the need to control abnormal cell cycle progression and to restrain tumor cell replication. Other groups documented that p21 mRNA and protein levels are reduced in HCC^83–85^. Since p21 is regulated by p53, it is believed that p21 expression in tumor cells depends on p53 expression status. As p21 inhibits DNA synthesis of human liver cancer cells, it is considered a target for HCC treatment^86^.

In this work, we have demonstrated that AM-CM treatment of hepatocarcinoma cells induces a significant increase in p53 and p21 expression and a downregulation in Mdm-2 expression, which could generate either an arrest in cell cycle or an increase in apoptosis entrance of tumor cells. Moreover, we have observed an increase in nuclear/cytoplasmic localization of p21 when HepG2 cells were treated with AM-CM. This observation is in concordance with the antiproliferative effect of AM-CM, since nuclear localization of 21 has been associated with its growth inhibitory function^87^. It has been demonstrated that p21 has dual functions depending on its cellular localization. Cytoplasmic p21 is related to the human cancer aggressiveness and its poor prognosis^88^. In the nucleus p21 causes growth arrest, senescence and cellular differentiation. On the other hand, in liver cirrhosis p21 correlates with the HCC outcome and is predominantly in cytoplasm when histology became more differentiated^89^. The inhibitory effect of p21 on G2/M cell cycle progression also correlates with its nuclear localization. Thus, AM-CM treatment would be effective to induce p21 nuclear localization promoting growth arrest of hepatocarcinoma cells.

The analysis of the cell cycle after treatment with AM-CM in HepG2 and HuH-7 cells revealed a higher number of cells in G2/M phase, whereas the number of cells in G0/G1 phase decreased compared with 0% FBS. The G2 checkpoint is regulated by the activation of diverse pathways that together inhibit the activity of the Cyclin B1/cdc2 kinase complex. P53 and p21 appear to be essential for maintaining the G2 checkpoint in human cells^90^. Our results demonstrated that the amniotic membrane conditioned medium generates a retardation in G2/M phase probably by suppressing the expression of Cyclin D1 and increasing p53 and p21, all together contributing to the amniotic membrane anticancer activity. Further studies will be needed to determine if the regulation of these signaling molecules by the AM-CM is also capable of triggering apoptosis in these cancer cells as another mechanism of tumor inhibition.

MicroRNAs (MiRs) are small non-coding RNA molecules of 21–24 nucleotides length that negatively regulate gene expression through translational repression or mRNA degradation, by binding to their target mRNA to its 3’untranslated region^91^. MiRs perform important roles in regulating gene expression and in consequence they are potential therapy targets in malignant cells. MiRs deregulation has been widely involved in human cancer and in particular in HCC. Some miRs contribute to tumor progression (oncoMiRs) and others inhibit its growth (antioncomiRs), and their identification could be significant for associated therapies in HCC patients. Between these miRs, miR-15a and miR-210 have been described with protumoral properties. MiR-15a expression has been found to be upregulated in liver cancer tissues associated with hepatitis B. Smad 7, which blocks TGF-β signaling, is a target of miR-15a in these tissues, leading to the suppression of cell growth^92^. MiR-210 has been reported to promote angiogenesis of liver cancers and in high levels is an indicator of HCC poor prognosis. It was also reported that miR-210 inhibition leads to proliferation arrest in HepG2 and HuH-7. MiR-210 has several targets that function to inhibit cell proliferation, such as E2F3, MYC antagonist (MNT) or ephrin-A3 (EFNA3)^92–94^.

MiR-206 has been reported to be significantly diminished in HCC tissues, and its overexpression can lead to apoptosis, cell cycle arrest, and proliferation, migration and invasion inhibition in HCC cells^95^. MiR-206 plays an important role in growth suppression in HCC cells. MiR-206 targets CDK-9, which is an important cyclin dependent kinase that stimulates production of prosurvival proteins. Thus, miR-206 downregulates CDK9 expression and inhibits tumor growth^96^. Another target of miR-206 is Cyclin D1, which has been described to be downregulated by this miR in lung tumor cells, decreasing cell proliferation^97^. Further experiments will be needed to determine if the increase in miR-206 expression is related to the decrease in Cyclin D1 expression in hepatocarcinoma cells. MiR-145 has been identified as a tumor suppressor in various types of cancer as it inhibits cell proliferation and cell invasion in tumor cells^98,99^. ROCK1 plays a central role in cell polarity, migration and chemotaxis and is elevated in several cancers. By targeting ROCK1, miR-145 diminishes tumoral cells invasion. In particular in HCC, miR-145 also has an antitumoral action since its overexpression is correlated with downregulation of proliferation, migration and invasion in hepatocarcinoma cell lines and in HCC tissues^100^. In agreement with these evidences, we have found that treatment with AM-CM induced an upregulation of antitumoral miR-206 and miR145, in HepG2 and HuH-7 hepatocarcinoma cells. On the contrary, AM-CM caused a significant downregulation of protumoral miR-15a and miR-210 in these cells, both at 24 and 72 h of treatment. Thus, the conditioned medium of the amniotic membrane has potential antitumoral properties since it would be able to regulate the miRs expression to control tumor growth. Additional experiments will be needed to determine other miRs regulation by AM-CM in HCC. In view of the need to find new and effective therapies for HCC treatment, here we have demonstrated that the conditioned medium isolated from human amniotic membranes has valuable antiproliferative properties: AM-CM was able to inhibit DNA synthesis, cell viability, and cell cycle progression involving a decrease in Ki-67 and Cyclin D1 expression, and the upregulation of p53 and p21 expression together with the modulation of pro and antitumoral miRs.

Particularly, the development of HCC pathogenesis is complex with possible crosstalks and redundancy in signaling pathways. Thus, the finding of therapies that covers the complex spectrum of signaling pathways and finally inhibits those that control tumor growth and survival will be probably success. The amniotic membrane anticancer properties are just starting to be discovered and they are promising. Further investigations will be performed to unravel the antitumor characteristics still unknown. Our results suggest that the amniotic membrane may be considered as a potential candidate for hepatocellular carcinoma treatments.

## Materials and Methods

### Ethics statement

Human placentas were handled as we previously described^8^. We obtained written informed consent from all subjects and all study procedures were approved by ethical review committees at the Alejandro Posadas National Hospital (Bioethics Comitte “Dr. Vicente Federico del Giudice”) and the Virgen Macarena University Hospital. All methods were carried out in accordance with relevant guidelines and regulations.

### Amniotic membrane conditioned medium preparation

After cesarean section from normal term pregnancies, human placentas (40 weeks) were obtained and transported to the laboratory suspended in ice-cold PBS.

The amnion membrane was processed as we previously described^8^. Briefly, amniotic membrane was manually stripped from the chorionic membrane, cut and placed in sterile physiological solution. The amnion was washed two or three times to completely remove bloody or torn pieces and cut into equal 4 pieces. These pieces were sterilized in a laminar flow through continuous washings in PBS containing 100 U/ml penicillin and 100 µg/ml streptomycin. Pieces from amnion were placed in 10-cm plates and incubated with DMEM-F12 0% FBS during 72 h. After remove the amnion membrane pieces, the remaining medium was centrifuged 10 min at 900 rpm to remove cellular debris. The cell pellet was discarded and conditioned medium was stored at −20 °C until cells treatment.

### Cell culture and treatment

The human hepatocarcinoma cell lines HepG2, Hep3B (Hep3B2.1-7), the breast cancer cell line MCF7, the melanoma cell line A375 and the choriocarcinoma cell line BeWo, were purchased from the American Type Culture Collection (ATCC, Rockville, MD). HuH-7 human hepatocarcinoma cell line was purchased from the Japanese Collection of Research Bioresources Cell Bank (JCRB, Japan). As we previously described^8^, cells were grown in Dulbecco Modified Eagle Medium (DMEM-F12) (Invitrogen) supplemented with 10% FBS (Gibco), 100 U/ml penicillin, 100 mg/ml streptomycin, 2 mM glutamine (Invitrogen), and 1 mM sodium pyruvate (Sigma) at 37 °C in 5% CO_2_.

In general, HepG2, HuH-7, Hep3B, MCF7, A375 and BeWo (1 × 10^6^) cells were grown during 24 h in complete DMEM-F12 10% FBS at 37 °C in 5% CO_2_. Then, medium was replaced with amniotic membrane conditioned medium (AM-CM) pure or diluted in a ratio 1:2 (1/2) or 1:4 (1/4) with DMEM-F12 0% FBS. In treatments corresponding to Fig. 1D,E, FBS (10%) was added to cells with the AM-CM. Cells cultured with 10% FBS or 0% FBS, were used as normal condition control or serum deprived control, respectively. Cells were treated during 24, 48 and 72 h.

### Quantitative real-time PCR assay (qRT-PCR)

TRISURE reagent was used according to manufacture instructions (Bioline Co) for total RNA extraction from control and treated HepG2 or HuH-7 cells

We performed qRT-PCR experiments as we previously described^8^. First, concentration and purity of the isolated RNA were estimated spectrophotometrically at 260 and 280 nm. For cDNA synthesis, 5 μg of total RNA was reverse transcribed at 50 °C during 1 h using the Transcriptor first Strand cDNA synthesis Kit (Roche). Quantitative RT-PCR reaction was performed using the primers forward and reverse specific for CYCLIN D1 (F: 5′-AGACCTTCGTTGCCCCTCGT-3′, R: 5′-CAGTCCGGGTCACACTTGAT-3′), p21 (F: 5′-GATGGCACCAGAGGTGGTTA-3′, R: 5′TCCCGAAATATTGGGGAAAG-3′), p53 (F: 5′-GGAAGAGAATCTCCGCAAGAA-3′, R: 5′-AGCTCTCGGAACATCTCGAAG-3′), MDM-2 (F: 5′-TTACCCAGGCTGGAGTGCAG-3′, R: 5′GAGAATGGTGCGAACCCG-3′), CYCLOPHILIN (F: 5′-CTTCCCCGATACTTCA-3′, R: 5′-TCTTGGTGCTACCTC-3′), and GAPDH (F: 5′-TCCCTGAGCTGAACGGGAAG-3′, R: 5′-GGAGGAGTGGGTGTCGCTGT-3′). QRT-PCR Master Mix Reagent kit (Fast Start universal SYBR Green, Roche) was used for PCR reactions that were performed on a Chromo 4 DNA Engine (Bio-Rad). One reaction contained 10 µM of forward and reverse primer, 2 µl of cDNA and the final reaction volume was 20 μl. The reaction consisted in preheating at 50 °C for 2 min, followed by heating at 95 °C for 10 min. Then, the amplification cycles (41) were carried out as follows: denaturation 15 sec at 95 °C and 1 min annealing and extension, at 60 °C. The Opticon Monitor 3 Program was used to determine the threshold cycle (CT) from each well. Relative quantification was calculated using the 2^−∆∆CT^ method^101^. For the treated samples, evaluation of 2^−∆∆CT^ indicates the fold change in gene expression, normalized to housekeeping genes (CYCLOPHILIN and GAPDH), and relative to the untreated cells (0% FBS). Specificity of amplifications was confirmed by melting curves analysis. Reaction mixtures without reverse transcriptase or RNA were run in parallel to ensure the absence of sample contamination.

### MicroRNA quantification by TaqMan MicroRNA assay

MicroRNA was reverse transcribed from total RNA isolated from HepG2 and HuH-7 cells, into a single stranded cDNA using the *Taq*Man^®^ MicroRNA Reverse Transcription Kit (ABI, USA) with specific primers for hsa-miR-15a, hsa-miR-210, hsa-miR-206, hsa-miR-145 and RNU6B (internal control for normalization). Relative expression of miR-15a, miR-210, miR-206, miR-145 was quantified using the TaqMan Real-Time *q*PCR (ABI Assay IDs: 002419 miR-15a, 000512 miR-210, 000510 miR-206, 000467 miR-145, 001093 RNU6B) with the StepOne™ Real-Time PCR System (ABI, USA). For the treated samples, evaluation of 2^−∆∆CT^ indicates the fold change in gene expression, normalized to internal control (RNU6B), and relative to the untreated cells (0% FBS).

### Western blot

We performed Western blot experiments as we previously described^8^. In brief, cells were incubated in 10-cm plates during different times, with DMEM-F12 10% FBS, 0% FBS or AM-CM (pure or diluted 1/2 or 1/4), as indicated in each Figure. Then, they were washed with 1X PBS. Samples were processed in lysis buffer and centrifuged to remove cellular debris. Bradford staining method^102^ was used to determine supernatant protein concentration (5μl), with BSA as standard. Lysates were mixed with Laemmli’s sample buffer containing 2% sodium dodecyl sulfate and 30 mM β-mercaptoethanol, boiled for 5 min in cracking buffer and resolved by SDS-PAGE on a 12% gel. Then, gels were electrophoretically transferred to a nitrocellulose membrane (Hybond; Amersham Pharmacia). Membranes were equilibrated in 1X PBS, and nonspecific binding sites were blocked by 3% BSA in PBS at room temperature for 30 min. Then they were immunoblotted with the specific antibody anti-p53 (mouse, 1:1000, Santa Cruz Biotechnology) or anti-Cyclin D1 (mouse, 1:1000, Santa Cruz Biotechnology). The antibodies were detected using horseradish peroxidase-linked goat anti-mouse IgG or anti-rabbit IgG (1:1000, Sigma), visualized by the Clarity Western ECL Substrate (Biorad) signaling system and an Amersham Imager 600 (GE Health Science). Control for equal gel loading was carried out by GAPDH (mouse, 1:5000, Santa Cruz Biotechnology) detection. Quantification of protein bands was determined by densitometry using Image J ink 1.45 program software (National Institute of Health, Bethesda, MD, USA).

### MTT assay

Metabolic activity was evaluated by the colorimetric test 3-(4,5-dimethylthia-zolyl-2) 2,5-diphenyltetrazolium bromide (MTT) (Sigma), as we previously described^8^. Cells that are metabolically active have dehydrogenase enzymes that can cleave tetrazolium ring of MTT and form dark blue formazan crystals that can subsequently be solubilized and quantified by spectrophotometry^103^. MTT solution (250 μg/ml) was added to all 24-wellss plate of an assay and cells were incubated at 37 °C during 30 min, at indicated times. In order to dissolve and extract the dark blue crystals ethanol 100% was added to all wells and mixed thoroughly. After a few minutes at room temperature, the plates were read on a micro ELISA 680x reader (Bio-Rad), at 570 nm. In parallel, the number of viable cells was determined by counting in a Neubauer chamber and the non-viable cells were excluded with trypan blue staining.

### UV irradiation

Before AM-CM treatment, HepG2 or HuH-7 cells were exposed to 40 J/m^2^ of UVC (5 sec) in a UV crosslinker CX 2000 (Thermofisher Scientific) according to Huang and Adamson^104^. The energy of the UV light was precisely controlled by the crosslinker. After exposure, the cells were grown in DMEM-F12 10% FBS, DMEM-F12 0% FBS or treated with AM-CM, for the indicated times. In control cells with DMEM-F12 10% FBS, UV treatment was omitted. Cells were then processed for MTT assay.

### Immunofluorescence staining

Immunofluorescence staining was performed as we previously described^8^. Briefly, after washing twice with PBS, HepG2 cells were fixed with 4% paraformaldehyde in PBS for 20 min at room temperature, at indicated times. Then, cells permeabilization with 0,1% Triton X-100 twice for 10 min, and blocking with 2% BSA were performed. Cells were incubated overnight with primary antibodies, including rabbit anti-p21 (1:100, Santa Cruz), rabbit anti-Ki-67 (1:100 Millipore) and mouse anti-Mdm-2 (1:100, Santa Cruz). Secondary antibodies Alexa Fluor 488-conjugated goat anti-rabbit IgG or Alexa Fluor 488-conjugated goat anti-mouse IgG (1:100, Thermo Fisher Scientific) were used according to the manufacturer’s instructions. The samples were incubated at room temperature for 1 h and washed 3 times with PBS. Nuclei were counterstained with DAPI, and samples were mounting with prolong Gold antifading solution (Thermoscientific). Primary antibody was omitted in negative controls (data not shown). Cells were visualized and photomicrographed under an inverted fluorescence microscope (Nikon). Positive Alexa 488 cells were analyzed and quantified using FIJI-Image J software (Bethesda, MD, USA).

### [H^3^]-Thymidine incorporation assay

HepG2 cells were grown in 10-cm plates (5 × 10^6^) in complete DMEM-F12 medium with 10% FBS. After 24 h, medium was replaced for the respective medium treatment (control 10% FBS, control 0% FBS or AM-CM), and cells were incubated for the indicated times. [^3^H]-thymidine incorporation assay was performed as we previously described^8^. In brief, 1 μCi/ml [^3^H]-thymidine (81 Ci/mmol) (Amersham Biosciences) was added 18 h before each end point. After washed three times with cold water, cells were harvested and centrifuged during 5 min. The cellular pellet was lysed with 5% TCA for 30 min, centrifuged, washed twice with cold PBS and resuspended in 150 μl 1 M NaOH for 1 h at room temperature. The incorporated radioactivity was quantified by a scintillation counter (Beckman) and DNA synthesis estimated as dpm/μg protein x h.

### Cell cycle analysis

HepG2 or HuH-7 cells (5 × 10^5^) were seeded in 6-wells plate in DMEM-F12 medium supplemented with 10% FBS, during 24 h. Next, medium was replaced for the different medium treatment (10% FBS control, 0% FBS control, AM-CM 1/2 or AM-CM 1/4). After 24 or 72 h, cells were washed twice with cold PBS and collected with trypsin-EDTA 0,05%. (Gibco) The cells were centrifuged for 5 min at 1500 rpm and then fixed in 70% cold ethanol overnight at −20 °C. Centrifugation was performed for 5 min at 1500 rpm at room temperature. The resulting cell pellet was treated with 10 μg/ml RNase A (Sigma) in PBS, at 37 °C for 30 min, and stained with 50 μg/ml propidium iodide (PI) (Sigma) during at least 2 h (RT) in the dark. The distribution of cells in different phases of cell cycle was examined using a BD FACSAria III flow cytometer (Becton Dickinson). The results were analyzed using the FlowJo software (Becton Dickinson, San José, USA). Each experiment was performed in triplicate.

### Data analysis

Statistical analysis were performed as we previously described^8^. Quantitative RT-PCR, MTT, cell count, cell cycle analysis and [H^3^]-thymidine incorporation assays were repeated separately at least three times to ensure reproducible results. For Western blots and immunofluorescence analysis, representative images of at least three independent experiments are shown along with quantification of immunoreactive bands and positive fluorescent cells, respectively. Results are expressed as the mean ± standard deviation (S.D.). The statistical significance was assessed by ANOVA followed by Bonferroni’s multiple comparisons post hoc test and was calculated using the GraphPad Instat computer program (GraphPad, San Diego,CA). A p value less than 0.05 was considered statistically significant.

## Supplementary information

Supplementary Figures

Dataset Figure 1, Dataset Figure 2, Dataset Figure 3,Dataset Figure 4
