# Supplementary material for: Human amniotic membrane conditioned medium inhibits proliferation and modulates related microRNAs expression in hepatocarcinoma cells
Source: Sci Rep. 2019 Oct 2;9:14193. doi: 10.1038/s41598-019-50648-5 (PMC6775050; doi:10.1038/s41598-019-50648-5)
Supplement: Supplementary file 1 — Supplementary Figures [file 41598_2019_50648_MOESM1_ESM.pdf]

Human amniotic membrane conditioned medium inhibits proliferation and modulates related microRNAs expression in hepatocarcinoma cells

Authors: Riedel, Rodrigo[1,2]; Pérez-Pérez, Antonio[3]; Carmona Fernández, Antonio[3]; Jaime, Mariana[4]; Casale, Roberto[4]; Dueñas, José Luis[5]; Guadix, Pilar[5]; Sánchez-Margalet, Víctor[3]; Varone, Cecilia L[1,2] and Maymó, Julieta L[1,2*].

Affiliation:
[1] Universidad de Buenos Aires. CONICET. Instituto de Química Biológica de la Facultad de Ciencias Exactas y Naturales (IQUIBICEN). Ciudad Universitaria Pabellón 2, 4º piso, (1428). Buenos Aires, Argentina.
[2] Universidad de Buenos Aires. Facultad de Ciencias Exactas y Naturales. Departamento de Química Biológica. Ciudad Universitaria Pabellón 2, 4º piso, (1428). Buenos Aires, Argentina.
[3] Departamento de Bioquímica Médica y Biología Molecular. Hospital Universitario Virgen Macarena. Facultad de Medicina. Universidad de Sevilla. Avenida Sánchez Pizjuán 4 (41009). Sevilla, España.
[4]Hospital Nacional Profesor Alejandro Posadas. Buenos Aires, Argentina
[5]Servicio de Ginecología y Obstetricia, Hospital Universitario Virgen Macarena. Sevilla, España

* To whom correspondence should be addressed:
Julieta Maymó
IQUIBICEN, CONICET; Departamento de Química Biológica, FCEN, UBA.
Ciudad Universitaria, Pabellón 2, piso 4
(1428), Buenos Aires, Argentina
Tell/fax: 54 11 4576 3342
E-mail: jmaymo@qb.fcen.uba.ar

Running title

Antitumoral properties of human amniotic membrane

Keywords

Amnion; antitumoral properties; hepatocarcinoma; human liver; cell survival.

Supplementary Figure 1

A

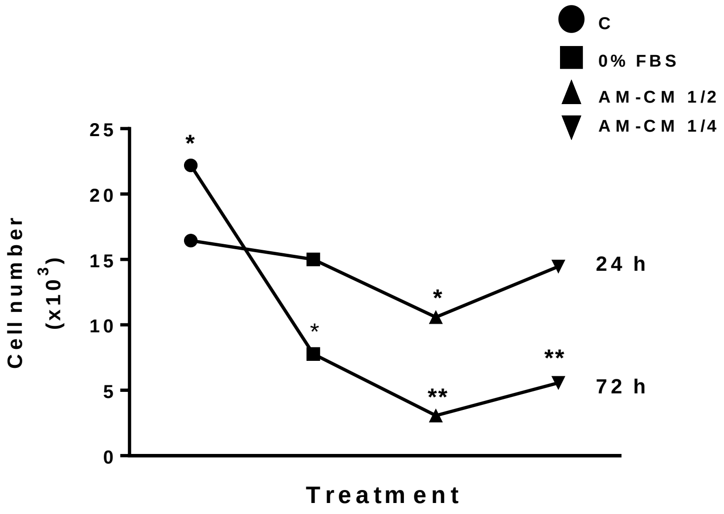

B

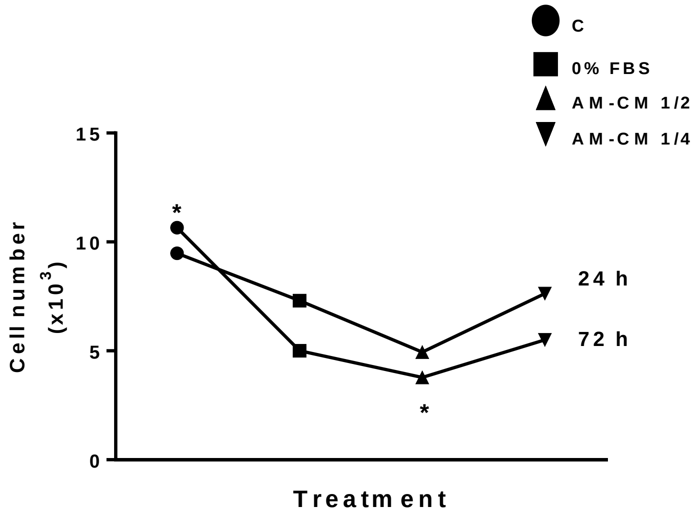

Supplementary Figure 1. *AM-CM reduces hepatocarcinoma cell number.* Dose responses of HepG2 (A) and HuH-7 (B) cell proliferation to AM-CM addition. Cells (500 000 cells/well) were grown in 6-well plates in complete DMEM-F12 media 10% FBS. After 24 h media were replaced by DMEM-F12 10% FBS (C), DMEM-F12 0% FBS, AM-CM 1/2 or AM-CM 1/4. Cells were cultured for 24 h or 72 h, at which cell count was performed, as indicated in Materials and Methods. Data are expressed as means ± S.D from four independent experiments. ANOVA was followed by Bonferroni analysis. *p<0.05, **p<0.01 vs. 24 h serum deprived control.

Supplementary Figure 2

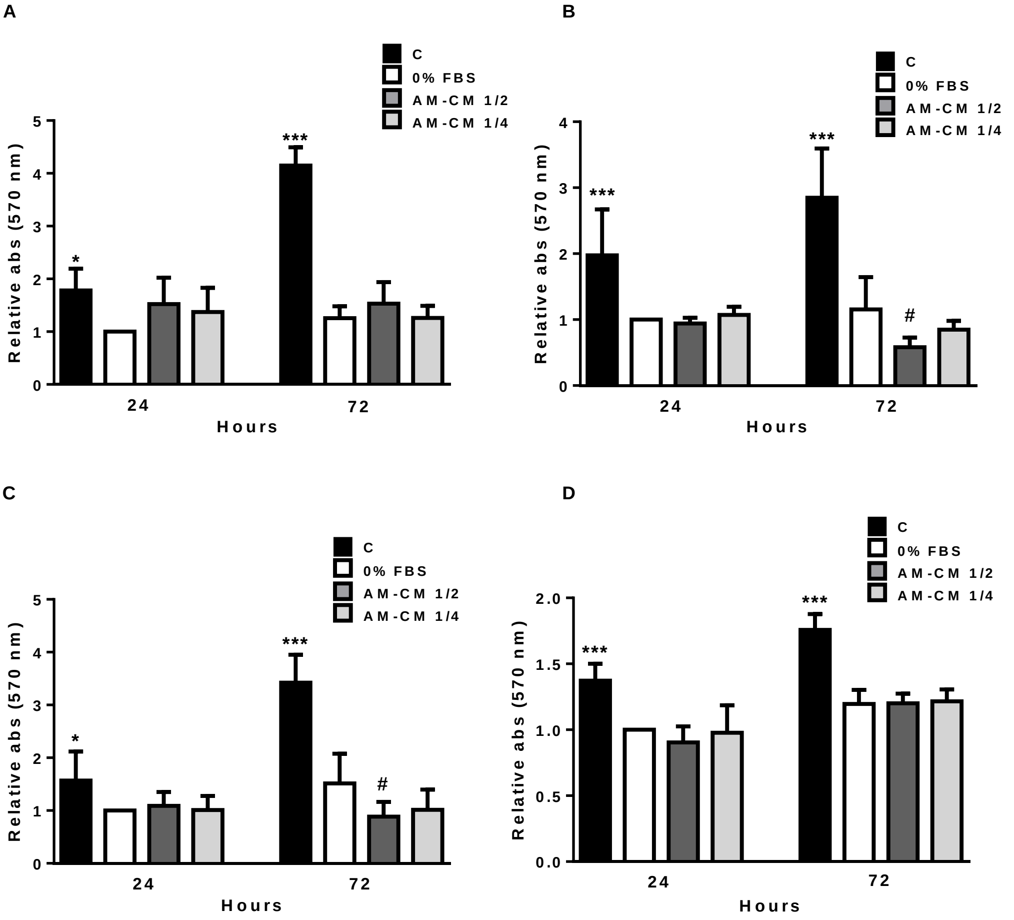

Supplementary Figure 2. *AM-CM antiproliferative effect is more suitable for HepG2 and HuH-7 hepatocarcinoma cells.* Hep3B cells **(A)**, A375 cells **(B)**, BeWo cells **(C)** and MCF-7 cells **(D)** were seeded in 24-well plates in complete DMEM medium supplemented with 10 % FBS (C) or in DMEM 0% FBS (0% FBS) or in AM-CM pure (AM-CM), diluted at 50% (AM-CM 1/2) or diluted at 25% (AM-CM 1/4). Cells viability was determined by MTT test at 24 h and 72 h of culture. Independent experiments were performed in duplicates four times. Results are expressed as means ± S.D. *p<0.05, ***p<0.01 vs. 24 h 0 % FBS control. #p<0.05 vs. 24 h 10% FBS control.

Supplementary Figure 3

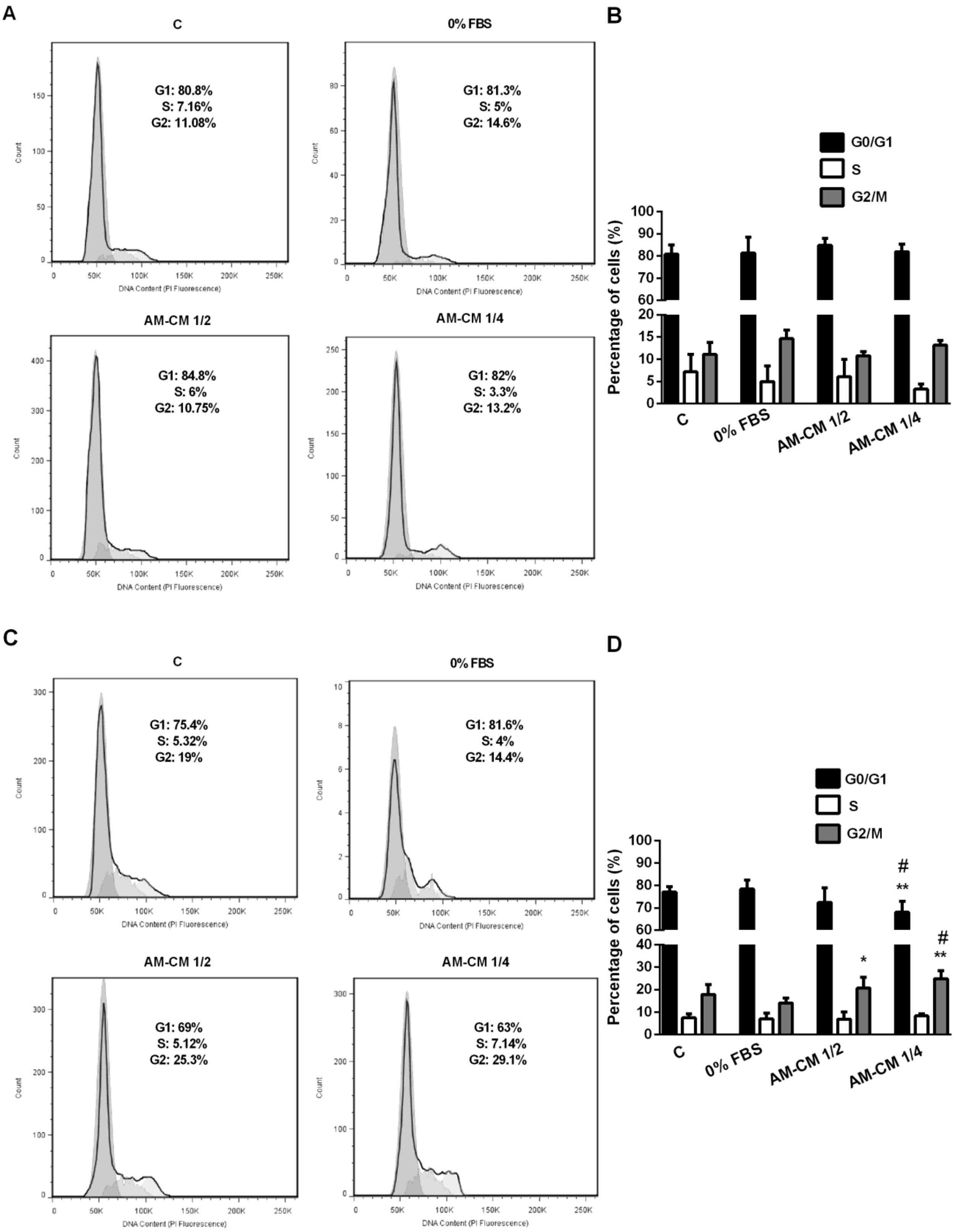

Supplementary Figure 3. *HuH-7 cells cycle progression is inhibited by AM-CM treatment.* HepG2 cells (5x105) were grown in 6-wells plate in DMEM-F12 10% FBS

during 24 h. Then, medium was replaced by treatment with DMEM 10% FBS (C), DMEM 0% FBS (0% FBS), AM-CM 1/2 or AM-CM 1/4. After 24h and 72 h cells were processed and analyzed by flow cytometry, as indicated in Materials and Methods. In **(A)** and **(C)** the cell population was selected on the basis of size (FSC-H) and granularity (SSC-H) and this population was plotted in PI-A vs PI-W plot in order to discriminate doublet population. Then singlet population was selected and plotted in count vs PI (propidium iodide) intensity graph. Graphs represent the distribution of HuH-7 cells in different phases of cell cycle at 24h **(A)** or 72 h **(C)** of treatment. The peak on the left in all graphs represents the G1 population and peak on the right represents the G2/M population. The valley between these two peaks represents the S phase population. **B-D.** The bar diagram represented the % of cells in different phases of cell cycle after 24 h **(B)** or 72 h **(D)** of treatment. Results are expressed as means ± S.D. (n=3) and one from a representative experiment is shown. **p<0.01 vs. 0% FBS; #p<0.05 vs. 10% FBS.

Supplementary Figure 4

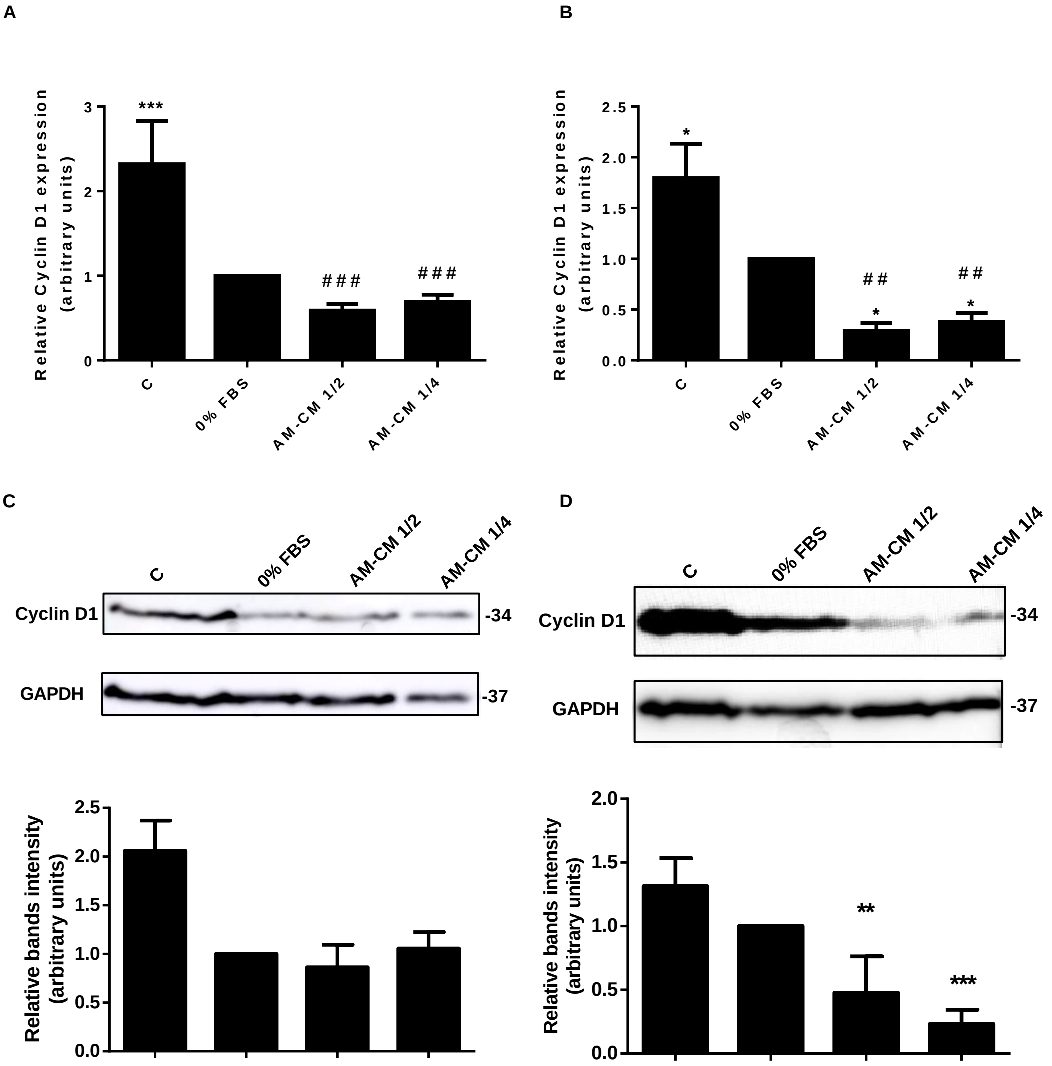

Supplementary Figure 4. *Cyclin D1 expression is downregulated by AM-CM treatment in HuH-7 cells.* HuH-7 cells were incubated with DMEM-F12 10% FBS (C), DMEM-F12 0% FBS, AM-CM 50% diluted (AM-CM 1/2), AM-CM 25% diluted (AM-CM 1/4) during 24 **(A)** or 72 h **(B)** before RNA extraction. Total RNA was extracted as described in Materials and Methods. CYCLIN D1 mRNA was measured by *q*PCR. CYCLOPHILIN and GAPDH were used as internal standards. **(C) (D)** HuH-7 cells were seeded in 10-cm plate and incubated with complete DMEM-F12 medium supplemented with 10% FBS (C), or without serum (0% FBS), or with AM-CM 50% (AM-CM 1/2) or AM-CM 25% (AM-CM 1/4). After 24 h **(C)** or 72 h **(D)**, cell extracts were prepared and proteins were separated on SDS-PAGE gels. Cyclin D1 expression in cell extracts was determined by Western blot. Molecular weights were estimated using standard protein markers. Loading controls were performed by immunoblotting the same membranes with anti-GAPDH. Lower panels show bands densitometry. Full-length blots are available in Supplementary Dataset. Molecular weight (kDa) is indicated at the right of the blot. **p<0.01, ***p<0.01 vs. 0% FBS; ##p<0.01, ###p<0.01 vs 10% FBS

Supplementary Figure 5

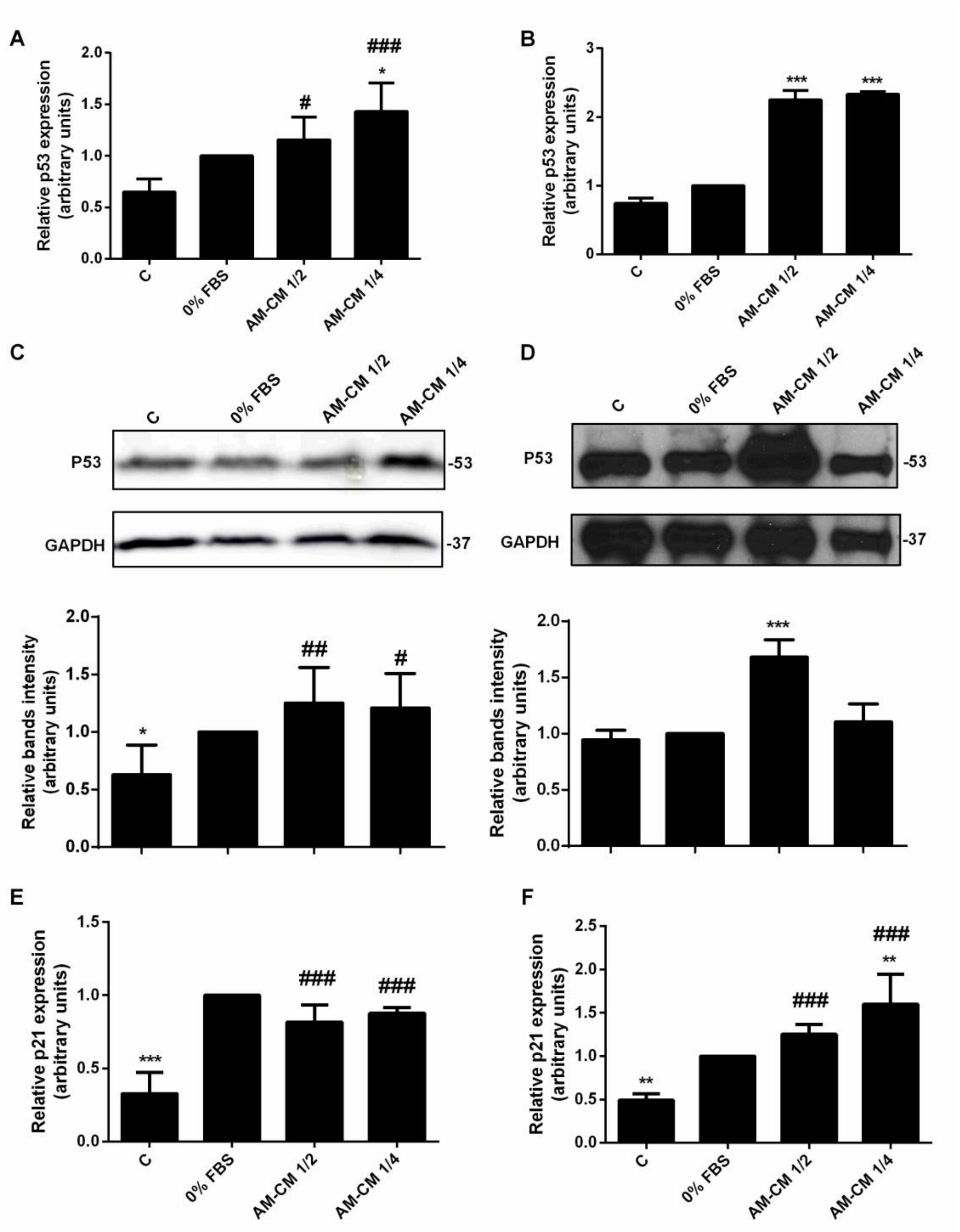

Supplementary Figure 5. *P53 and p21 expression increases in HuH-7 cells after AM-CM treatment.* HuH-7 cells were plated in complete DMEM-F12 medium 10 % FBS (C), in DMEM-F12 0% FBS (0% FBS), in AM-CM diluted at 50% (AM-CM 1/2) or at 25% (AM-CM 1/4) and incubated during 24 h **(A)** or 72 h **(B)** before total RNA extraction. P53 mRNA was measured by *q*RT-PCR. CYCLOPHILIN and GAPDH were used as internal standards. **(C) (D)** HuH-7 cells were seeded in 10-cm plate and incubated with complete DMEM-F12 10% FBS (C), or without serum (0% FBS), or with AM-CM 50% (AM-CM 1/2) or AM-CM 25% (AM-CM 1/4). Cell extracts were prepared at indicated times and proteins were separated on SDS-

PAGE gels. P53 expression at 24 h **(C)** or 72 h after treatment **(D)** was determined by Western blot. Molecular weight was estimated using standard protein markers and is indicated at the right of the blot. Loading controls were performed by GAPDH detection. Bands densitometry is shown in lower panels. Full-length blots are available in Supplementary Dataset. **(E) (F)** HuH-7 cells were incubated with 10 % FBS (C), DMEM-F12 0% FBS (0% FBS), AM-CM 1/2, AM-CM 1/4 during 24 h **(E)** or 72 h **(F)** before RNA extraction. Total RNA was extracted as described in Materials and Methods. P21 mRNA was measured by quantitative real time PCR. CYCLOPHILIN and GAPDH were used as internal standards. Results from a representative experiment are shown and expressed as means ± S.D. for five independent experiments performed in duplicates. For Western blot, representative results from three replicates are shown. *p<0.05, **p<0.01, ***p<0.001 vs. 0% FBS; #p<0.05, ##p<0.01, ###p<0.001.
