# Supplementary material for: Human amniotic membrane conditioned medium inhibits proliferation and modulates related microRNAs expression in hepatocarcinoma cells
Source: Sci Rep. 2019 Oct 2;9:14193. doi: 10.1038/s41598-019-50648-5 (PMC6775050; doi:10.1038/s41598-019-50648-5)
Supplement: Supplementary file 2 — Dataset Figure 1, Dataset Figure 2, Dataset Figure 3,Dataset Figure 4 [file 41598_2019_50648_MOESM2_ESM.pdf]

Human amniotic membrane conditioned medium inhibits proliferation and modulates related microRNAs expression in hepatocarcinoma cells

Authors: Riedel, Rodrigo[1,2]; Pérez-Pérez, Antonio[3]; Carmona Fernández, Antonio[3]; Jaime, Mariana[4]; Casale, Roberto[4]; Dueñas, José Luis[5]; Guadix, Pilar[5]; Sánchez-Margalet, Víctor[3]; Varone, Cecilia L[1,2] and Maymó, Julieta L[1,2*].

Affiliation:
[1] Universidad de Buenos Aires. CONICET. Instituto de Química Biológica de la Facultad de Ciencias Exactas y Naturales (IQUIBICEN). Ciudad Universitaria Pabellón 2, 4º piso, (1428). Buenos Aires, Argentina.
[2] Universidad de Buenos Aires. Facultad de Ciencias Exactas y Naturales. Departamento de Química Biológica. Ciudad Universitaria Pabellón 2, 4º piso, (1428). Buenos Aires, Argentina.
[3] Departamento de Bioquímica Médica y Biología Molecular. Hospital Universitario Virgen Macarena. Facultad de Medicina. Universidad de Sevilla. Avenida Sánchez Pizjuán 4 (41009). Sevilla, España.
[4]Hospital Nacional Profesor Alejandro Posadas. Buenos Aires, Argentina
[5]Servicio de Ginecología y Obstetricia, Hospital Universitario Virgen Macarena. Sevilla, España

* To whom correspondence should be addressed:
Julieta Maymó
IQUIBICEN, CONICET; Departamento de Química Biológica, FCEN, UBA.
Ciudad Universitaria, Pabellón 2, piso 4
(1428), Buenos Aires, Argentina
Tell/fax: 54 11 4576 3342
E-mail: jmaymo@qb.fcen.uba.ar

Running title

Antitumoral properties of human amniotic membrane

Keywords

Amnion; antitumoral properties; hepatocarcinoma; human liver; cell survival.

Supplementary Data Figure 1

a

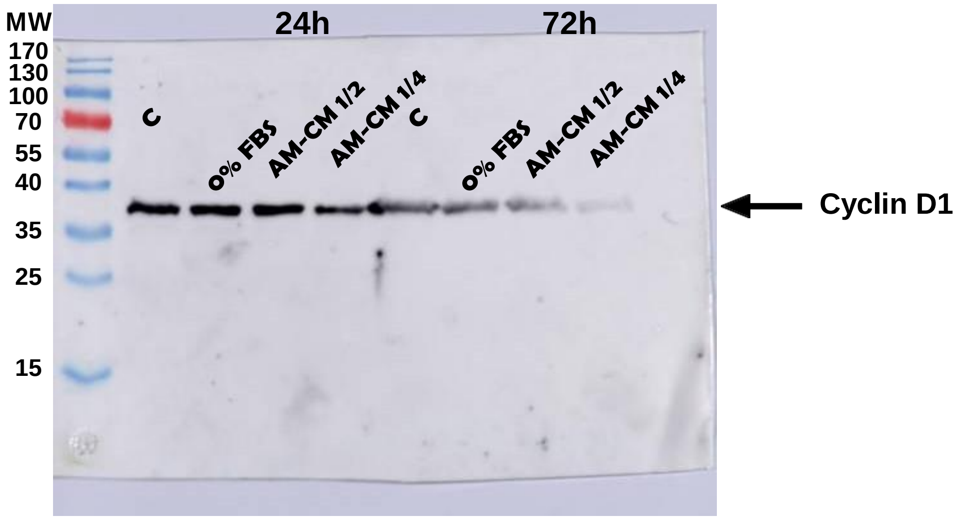

b

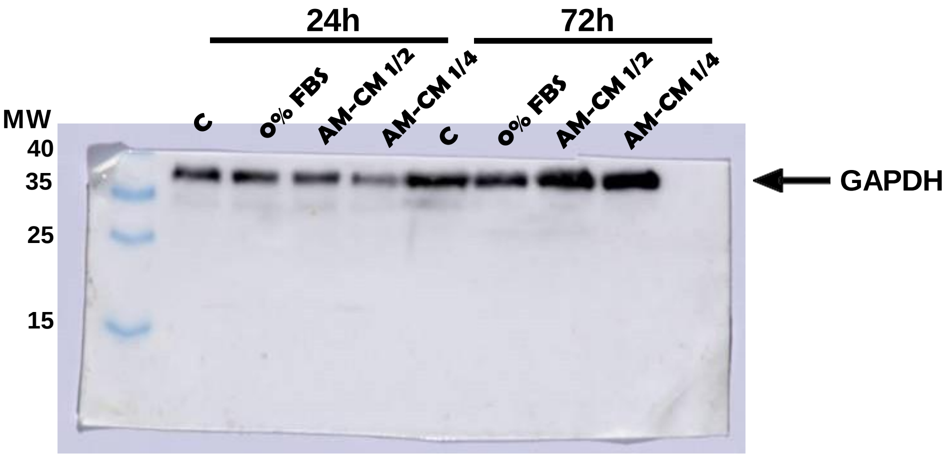

Supplementary Data Figure 1

Full-length blots corresponding to crops showed in Figures 3C and 3D. (a) Cyclin D1 (b) GAPDH.

Supplementary Data Figure 2

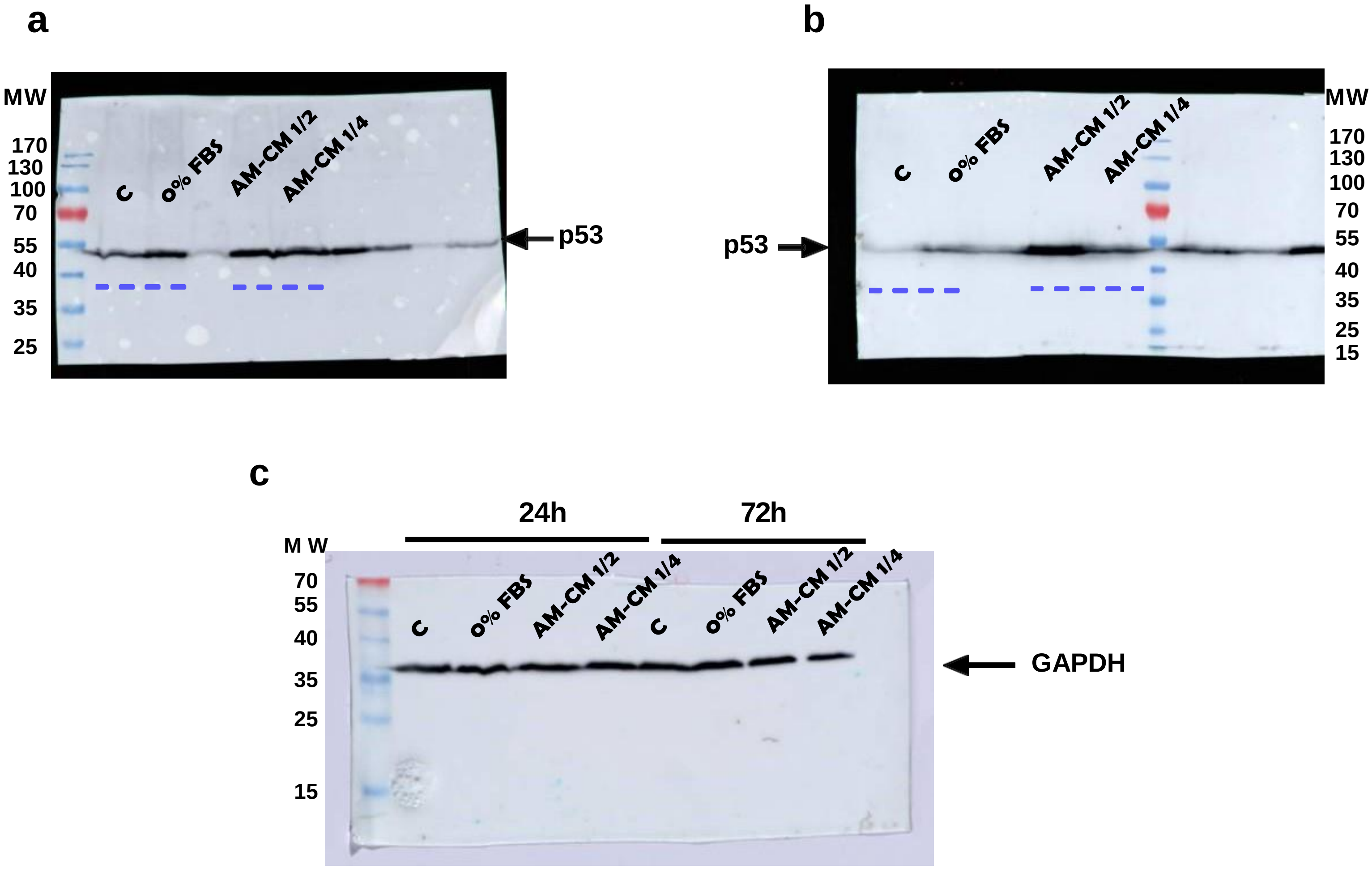

Supplementary Data Figure 2

Full-length blots corresponding to crops showed in Figures 5C and 5D. (a) p53 (b) GAPDH. Dashed blue line indicates the portion of the blot that was used in the figure.

Supplementary Data Figure 3

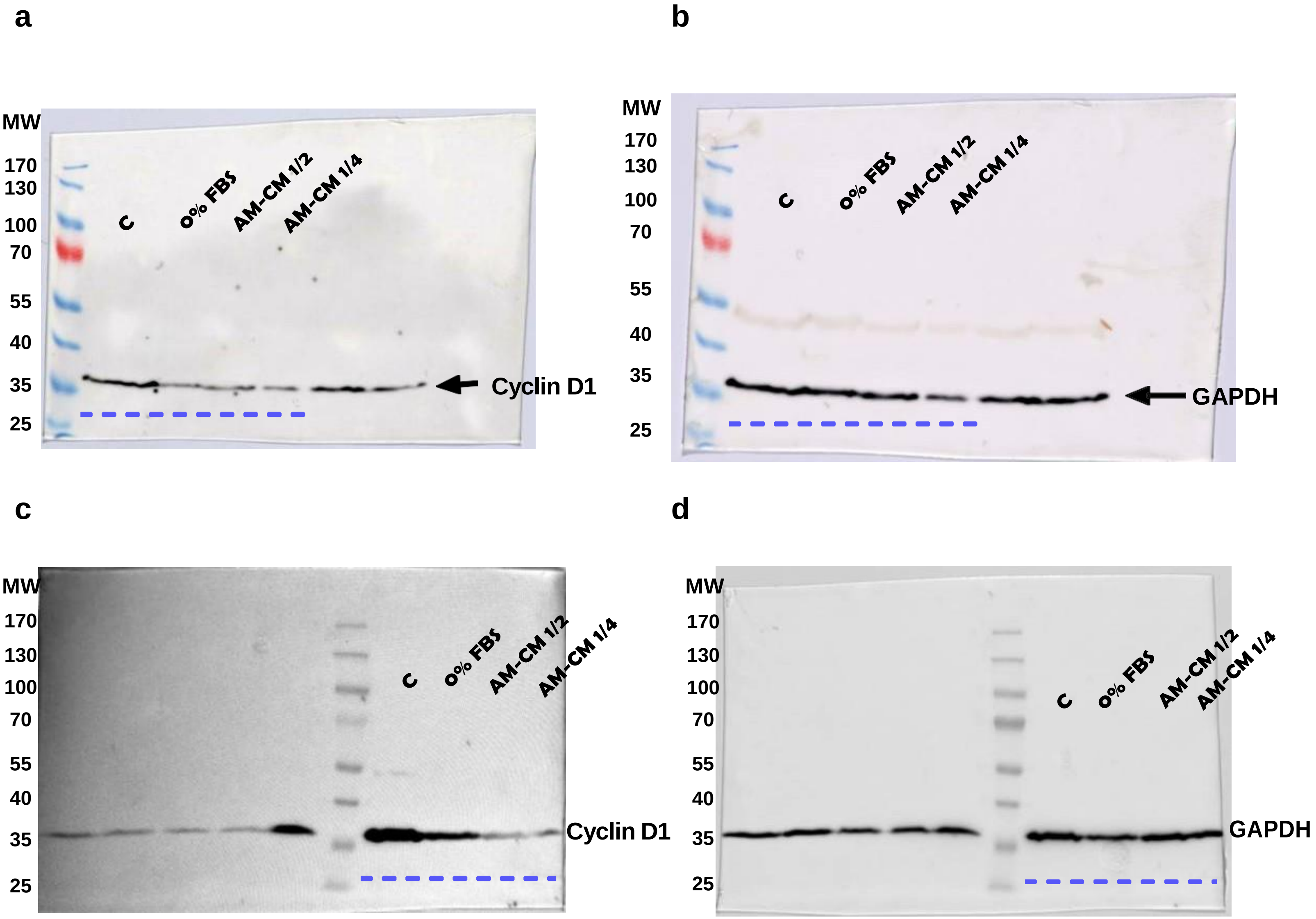

Supplementary Data Figure 3

Full-length blots corresponding to crops showed in Supplementary Figures 4C and 4D (a) Cyclin D1 (24h), (b) GAPDH (24h), (c) Cyclin D1 (72h) and (d) GAPDH (72h). Dashed blue line indicates the portion of the blot that was used in the figure.

Supplementary Data Figure 4

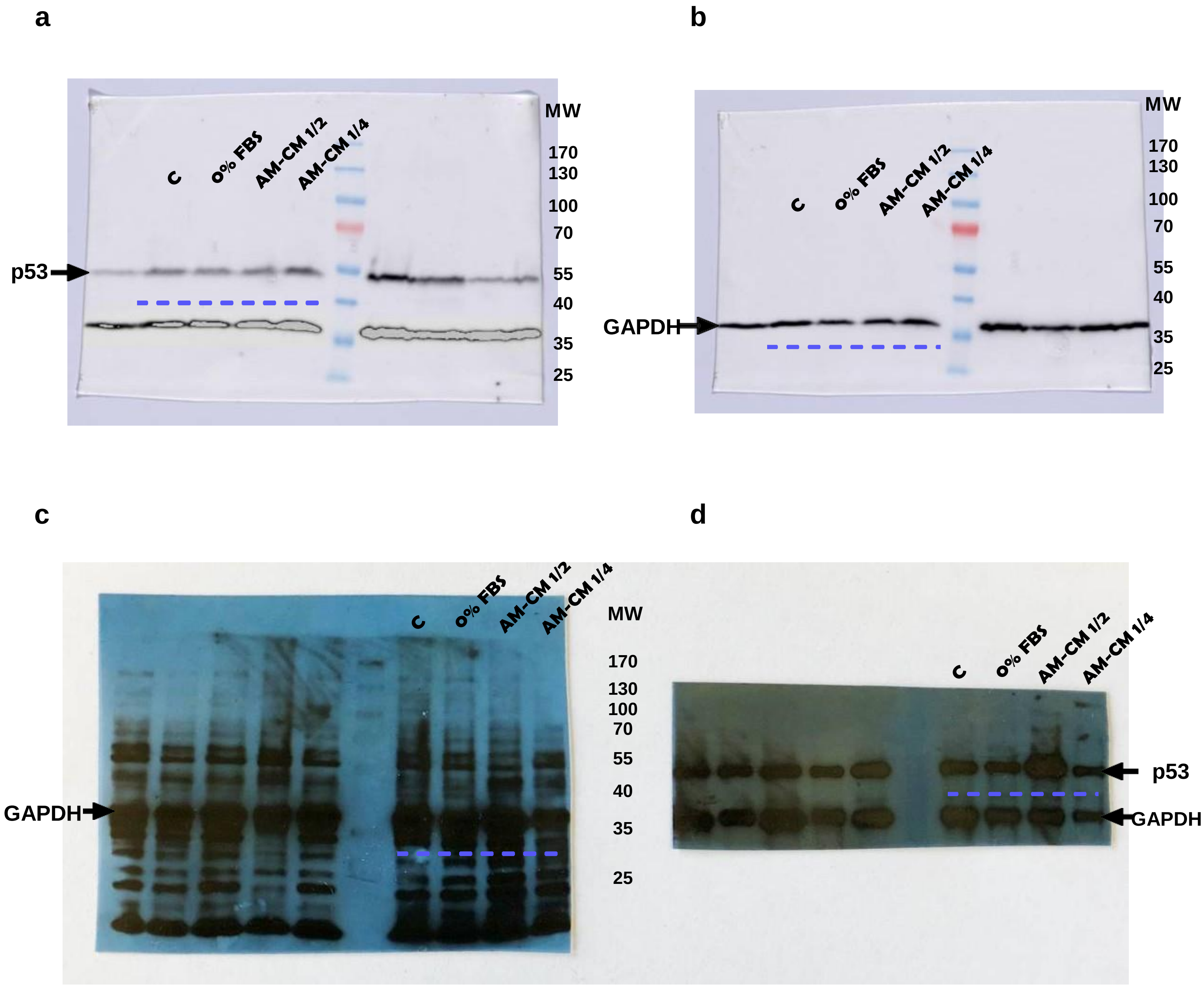

Supplementary Data Figure 4

Full-length blots corresponding to crops showed in Supplementary Figures 5C and 5D. (a) p53 (24h), (b) GAPDH (24h), (c) GAPDH (72h) and (d) p53 and GAPDH (72h). Dashed blue line indicates the portion of the blot that was used in the figure.
